# FedTKD: A Trustworthy Heterogeneous Federated Learning Based on Adaptive Knowledge Distillation

**DOI:** 10.3390/e26010096

**Published:** 2024-01-22

**Authors:** Leiming Chen, Weishan Zhang, Cihao Dong, Dehai Zhao, Xingjie Zeng, Sibo Qiao, Yichang Zhu, Chee Wei Tan

**Affiliations:** 1 School of Computer Science and Technology, China University of Petroleum (East China), Qingdao 266580, China; chenleiming2020@163.com (L.C.); zhangws@upc.edu.cn (W.Z.); dch142857@163.com (C.D.); xxz20181027@163.com (Y.Z.); 2 CSIRO’Data61, Sydney 2015, Australia; dehai.zhao@data61.csiro.au; 3 School of Computer Science, Southwest Petroleum University, Chengdu 610500, China; zengxjupc@163.com; 4 School of Software, Tiangong University, Tianjin 300387, China; 5 School of Computer Science and Engineering, Nanyang Technological University, Singapore 639798, Singapore

**Keywords:** heterogeneous federated learning, adaptive knowledge distillation, malicious client identification, trustworthy knowledge aggregation

## Abstract

Federated learning allows multiple parties to train models while jointly protecting user privacy. However, traditional federated learning requires each client to have the same model structure to fuse the global model. In real-world scenarios, each client may need to develop personalized models based on its environment, making it difficult to perform federated learning in a heterogeneous model environment. Some knowledge distillation methods address the problem of heterogeneous model fusion to some extent. However, these methods assume that each client is trustworthy. Some clients may produce malicious or low-quality knowledge, making it difficult to aggregate trustworthy knowledge in a heterogeneous environment. To address these challenges, we propose a trustworthy heterogeneous federated learning framework (FedTKD) to achieve client identification and trustworthy knowledge fusion. Firstly, we propose a malicious client identification method based on client logit features, which can exclude malicious information in fusing global logit. Then, we propose a selectivity knowledge fusion method to achieve high-quality global logit computation. Additionally, we propose an adaptive knowledge distillation method to improve the accuracy of knowledge transfer from the server side to the client side. Finally, we design different attack and data distribution scenarios to validate our method. The experiment shows that our method outperforms the baseline methods, showing stable performance in all attack scenarios and achieving an accuracy improvement of 2% to 3% in different data distributions.

## 1. Introduction

As deep learning continues to advance, many organizations use artificial intelligence technology to optimize their digital management processes. Nevertheless, while artificial intelligence brings undeniable benefits, it has ushered in a new set of challenges. First and foremost, the widespread adoption of cross-industry data analysis methodologies has transformed data analysis into a collaborative, multidisciplinary endeavor. This shift means that data analysis no longer confines itself to a single industry but rather spans various sectors. Consequently, the need to pool data resources from diverse domains has become increasingly apparent. This collaborative approach extends to constructing model training datasets, necessitating cooperation among institutions within the same industry and across different sectors. One solution to addressing these challenges is multi-party data sharing. However, implementing this approach introduces its own set of issues. Traditional data analysis practices entail the collection of data from multiple sources, centralizing it on a server for analysis. Unfortunately, this centralized data collection method poses a significant risk to data privacy. Furthermore, in certain sectors such as healthcare and finance, where data often contains sensitive personal or proprietary information, direct industry data sharing becomes infeasible. Consequently, the need to achieve multi-party federated data analysis while preserving data privacy has become an urgent concern. Simultaneously, the demand for complex tasks has driven organizations to train larger network models with an increased number of parameters. These large, highly accurate models have successfully addressed challenges in various domains. However, resource constraints and associated costs have made it challenging for some participants to engage in the training of such complex models. This predicament has placed the spotlight on addressing model training for resource-constrained participants.

To tackle the data privacy conundrum on mobile devices, Google pioneered federated learning technology [1]. Federated learning is a distributed computing method that solves data privacy issues by exchanging model parameters between mobile devices and servers instead of sharing raw data. Google also proposed the FedAvg method [1], a global fusion method that calculates the client weights based on the ratio of client samples to the total samples. However, in practical scenarios, this method faces two significant challenges. On the one hand, the inherent diversity in devices and data sources makes it difficult to achieve convergence in the fused global model when using the average weighting method employed by FedAvg. On the other hand, frequent model transfers between the server and clients result in increased communication costs, which can be unacceptable in resource-constrained wireless and mobile network environments. Therefore, some research [2,3,4] has concentrated on enhancing model convergence speed and optimizing communication costs in federated learning. Despite these efforts to address model convergence in federated learning, most of these methods assume a uniform network model and structure across all clients. In practice, different clients may design their own network models to suit their computational environments, giving rise to the “heterogeneous model fusion” challenge in federated learning. Conventional federated learning algorithms are ill-equipped to resolve this challenge.

To address the constraints imposed by computationally limited devices, Hinton et al. introduced knowledge distillation [5]. This technique guides the training of a small model (student model) on resource-constrained devices by first pre-training a complex network model (teacher model) with high performance. Knowledge is then transferred from the teacher model to the student model, enabling the latter to perform better. Notably, knowledge distillation permits the teacher and student models to possess different network structures, effectively solving the problem of knowledge transfer between heterogeneous networks. Leveraging the advantages of knowledge distillation, researchers have explored its integration with federated learning, which brings the series of federated knowledge distillation methods. Federated knowledge distillation, a two-step knowledge distillation process encompassing server-side and client-side knowledge distillation, has emerged as a solution to the heterogeneous model fusion problem in federated learning. However, the combination also brings some new challenges.

On the one hand, in the server-side knowledge distillation phase, each client model assumes the role of a teacher, while the server-side model serves as the student. Allocating the fusion weight of each student is the key issue. Some research endeavors implement multi-teacher knowledge distillation by maintaining fixed teacher weights. Moreover, certain approaches employ rotating teacher selection [6] to facilitate knowledge distillation. These methods address the challenge of knowledge fusion in heterogeneous models but do not entirely resolve the issue of knowledge distillation in federated learning. Federated learning, characterized by dynamic changes in the client model with each communication round, necessitates a method that can dynamically select the teacher model based on the quality of the client model. Furthermore, each federated learning communication round involves distributing server-side knowledge to individual clients, posing challenges in leveraging global knowledge to guide the training of each client’s private model.

On the other hand, we cannot share clients’ private data in the federated learning environment, so training a teacher model while preserving client privacy remains the new issue. Current research in this direction can be broadly categorized into two main approaches: the public dataset method [7,8,9] and the method based on Generative Adversarial Networks (GANs) [10,11,12]. Nevertheless, the GAN-based method requires clients to possess significant computational resources, and GAN training is a time-consuming process, limiting its accessibility to some participants. The public dataset method, too, faces its own set of challenges. It assumes the trustworthiness of all participating clients, but in reality, some clients may exhibit malicious behavior. These clients may intentionally manipulate the outputs of their local models on public datasets. Incorporating malicious information into global knowledge fusion can significantly impact overall accuracy. Additionally, variations in each client’s computational power and data quality result in differences in the quality of information output by client models on public datasets. Some algorithms (e.g., FedMD [7]) employ an average weighting method to merge this information, leading to compromised global information performance. While current approaches, such as public datasets and GAN-based methods, offer partial solutions to the problem of heterogeneous federated learning, they presume the trustworthiness of all federated learning participants. However, some clients may produce malicious or low-quality knowledge in real-world environments, making achieving trusted knowledge aggregation in heterogeneous settings challenging. For instance, in medical image applications, certain hospitals have designed personalized models tailored to their specific computational environments. These hospitals seek to reuse their original models and collaboratively train a more accurate model based on their existing models. Before embarking on the federated task, these hospitals must annotate their respective sample data to prepare the training dataset. Medical dataset annotation is a complex task that demands domain expertise and significant time investment. Annotators’ domain knowledge directly impacts the quality of image annotations. Errors in annotation may result in discrepancies in the knowledge output by each hospital’s model. Additionally, in an attempt to reduce costs or subvert the co-trained models, some individuals may intentionally upload entirely random knowledge or employ randomly labeled samples for model training, generating malicious knowledge. Utilizing such malicious or low-quality knowledge for global knowledge fusion can jeopardize the entire federated task.

These challenges make building a framework for Trustworthy AI increasingly imperative. In efforts to promote Trustworthy AI applications, Lu et al. have explored various domains, including software development processes [13] and pattern design [14]. Trustworthy federated learning technology plays a pivotal role in safeguarding privacy and enabling collaborative learning for Trustworthy AI. To actualize Trustworthy AI, Chen et al. [15] have devised a computational framework for trustworthy federated learning, ensuring the security of the entire federated learning process.

In response to these challenges, we propose a knowledge distillation-based approach that utilizes public data to address the problem of heterogeneous model fusion in federated learning. Our objectives encompass (1) achieving trustworthy global information fusion in heterogeneous federated learning environments and (2) enhancing the accuracy of knowledge transfer between servers and clients through knowledge distillation techniques. To attain these goals, we present a trustworthy federated learning framework grounded in knowledge distillation. This framework incorporates client information verification on the server side to prevent malicious clients from participating in information fusion. Additionally, we introduce a model training methodology based on two-stage knowledge refinement to elevate the accuracy of client models.

The main contributions of this paper are as follows:

- We design a trustworthy federated learning framework in a heterogeneous environment, which can realize client identification and trustworthy knowledge fusion.
- We propose a malicious client identification method based on client logit features. To the best of our knowledge, we are the first to propose a malicious client identification method based on logit information in heterogeneous federated learning.
- We propose a trustworthy global logit computation method, which ensures the accuracy of global logit information by training the model on the server side. At the same time, it can adaptively fuse the logit information of each client to realize a high-quality fusion of logit information.
- We propose an adaptive knowledge distillation method, which can adaptively select the parameters of knowledge distillation according to the confidence of the server-side global logit, and the method can improve the accuracy of the client model.
- We design different attack scenarios to verify the reliability of our methods. Meanwhile, we compare five baseline algorithms under different data distribution scenarios to verify the performance of our approach.

## 2. Related Work

### 2.1. Federated Learning

The FedAvg method, initially proposed by Google [1], computes fusion weights for each client model based on the ratio of client samples to the total. However, due to device and data heterogeneity, FedAvg may lead to slow global model convergence. Therefore, several research focuses on improving convergence problem, such as the FedProx algorithm [3], which introduced a loss function that corrects the parameters of the clients’ models that deviate from the global model, thus accelerating the convergence of the global model. Karimireddy et al. also proposed the Scaffold algorithm [2], which corrects the client drift problem by introducing control variables. Wang et al. proposed the FedNova algorithm [16]. This method requires each client to upload normalized model parameters, and then the server calculates the average parameters, thus solving the global model fusion problem. Li et al. proposed the MOON algorithm [17], which solves the global model convergence problem by introducing the contrast loss of the model.

Although these methods solve the global model convergence problem, they require clients to use the same structural model, so they cannot work in a heterogeneous model environment, making it challenging to perform federated learning in a heterogeneous environment.

### 2.2. Federated Knowledge Distillation

Knowledge distillation can transfer knowledge between heterogeneous networks. Some research has applied knowledge distillation to federated learning scenarios to solve the fusion problem of heterogeneous models. However, we cannot share participants’ private data in the federated learning environment. How to train teacher models while guaranteeing privacy becomes an urgent problem. We can categorize this research into three primary categories: public dataset-based, calculating category information, and using data-generated approaches.

The public dataset-based approach accomplishes client-side and server-side knowledge distillation by constructing a public dataset between the server and the client. For example, Li et al. proposed FedMD [7], which supports using different models for each client and accomplishes heterogeneous model knowledge distillation through the public dataset. Lin et al. proposed the FedDF method, which aggregates the average soft-label information uploaded by the clients to the server side and then computes the average soft-label information sent to the client. Jiang et al. proposed the FedDistill method [9], which builds a personalized model for each client and transfers the knowledge from the global model to the personalized model. Some researchers use the category information of the client’s private dataset to transfer knowledge. For example, the FedDistill+ method [18] requires each client to calculate the average logit information of each category, and the server-side fuses this information to form the global logit information of each category and finally guides the client model training through this information. Chan et al. proposed the FedHe method, which also aggregates the logit information of private data to achieve knowledge aggregation and then uses this knowledge to guide the model training of each client. Chen et al. also proposed FedHKD [19], which sends the client’s data representations and corresponding soft predictions to the server, and the server aggregates this information to achieve knowledge merging. Generative model-based methods mainly solve the knowledge fusion problem by training GAN networks to generate client data samples. For example, Zhu et al. proposed the FedGEN [10] method to achieve client-side model aggregation by generating a lightweight data generator on the server side. Such as, Zhang et al. proposed the FedFTG method [11] to transfer knowledge from local models to global models by exploring the input space of local models through generators. The FedDTG method [12] implements knowledge fusion by training GAN networks to generate pseudo-samples.

Although the GAN method solves the problem of client data protection, training GAN requires the client to have high computational power, and the training process of GAN is very time-consuming. Therefore, some resource-limited clients cannot use this method. Although the public dataset approach can use fewer resources to train teacher models, the current method cannot guarantee trustworthy federated learning, which needs an evaluation step for client information. The private data-based approach does not require the construction of additional datasets. However, the method uses the average knowledge of each category to guide the client model training, which is only effective on very simple data sets. Therefore, achieving a credible heterogeneous federated learning algorithm is important while maintaining the efficiency and accuracy of knowledge calculation.

### 2.3. Trustworthy Federated Learning

Current research aims to achieve credible federated learning in three main categories: client identification, model parameter-based identification, and model parameter pruning method. The client selection method prevents malicious clients from participating in fusion by identifying their behavior. For example, Li et al. proposed a reliable federated learning framework [20], which uses a spectral anomaly detection method on the server side to realize the identification of the malicious client. Chen et al. also proposed a reinforcement learning method to adaptively select the trusted clients for model aggregation [21]. Model parameter identification-based methods mainly distinguish malicious clients by calculating the Euclidean distance between models, such as Krum and Mutil-Krum [22]. In addition, Chen et al. [23] also proposed a model fusion feature approach to identify malicious clients. Model parameter pruning-based methods achieve secure global model fusion by pruning the outlier parameters of malicious models. These methods include TrimmedMean [24], ClippedClustering [25], and Centered Clipping [26] methods.

Although these methods address the problem of trustworthy federated learning, most research is based on evaluating clients’ model accuracy or detecting models’ parameters. However, each client may have a different model structure in the heterogeneous federated learning scenario. Therefore, these methods cannot solve the problem of heterogeneous model detection. Some federated learning algorithms use model logit on datasets in heterogeneous environments for global information fusion. Therefore, using the logit information to detect malicious information becomes the key to solving this problem. Zhang et al. proposed a credible federated learning framework (RobustFL) [27], which realizes the identification of malicious clients by constructing a predictive model based on logit on the server side. Wang et al. also proposed a method based on the distribution of logit to determine the distribution of malicious samples [28].

However, these methods encounter the cold-start problem because they must collect specific malicious logit information and train a predicted model before detecting malicious information. The malicious model outputs different information in each communication round. We cannot train a recognition model in every communication round, which will affect the progress of federated learning. Therefore, we need a real-time dynamic detection method for malicious message identification.

## 3. Method

### 3.1. Problem Definition

In real federated learning environments, there are differences in computing resources and computing environments across clients, leading to clients needing to design their respective models according to their environments and problems. Therefore, each client may use a different network structure, known as the model heterogeneity problem. Traditional federated learning algorithms (e.g., FedAvg, FedPorx, etc.) require clients to use the same model and structure. These methods ensure that the parameters of each layer of the model are consistent, so the server-side computes the global model parameters by fusion of the model parameters of each layer. However, these methods cannot be used in heterogeneous federated learning. To describe the heterogeneous model problem, we will describe the critical processes of heterogeneous model fusion in federated learning and the problems in the process. The knowledge distillation method is a common method to solve the information transfer of heterogeneous networks. Therefore, we also use the knowledge distillation method to solve the fusion problem of heterogeneous models in federated learning.

#### 3.1.1. The Processes of Federated Knowledge Distillation

**Figure 1:**
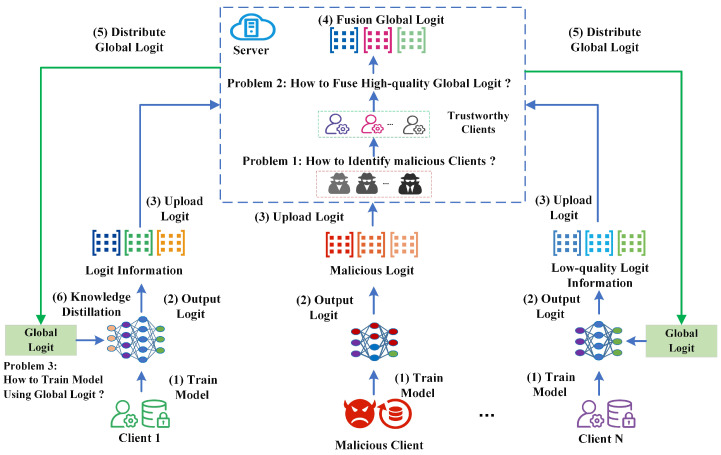
Heterogeneous network fusion method based on logit information.

Traditional federated learning approaches accomplish information transfer by passing models between the server and the client. In a federated heterogeneous modeling environment, the structure of each client’s model is different, which makes it unable to use the traditional approach. To complete the information transfer between the client and the server side, we exchange information through the logit information output from the model. The method is divided into six steps, as shown in Figure 1, where the key processes are described as follows.

**(1) Train Local Model**: The client trains the local model on the private dataset and then uses the local model to output the logit information on the private or public dataset.

**(2) Output Logit Information**: Different federated knowledge distillation methods require different logit information output. For example, public dataset-based knowledge distillation methods need to share a public dataset between server and client, and these methods require each client to upload each sample’s logit of the public dataset. However, private dataset-based methods only need to upload the logit information of the private dataset of each client (e.g., the average logit information of the client-side categories).

**(3) Global Logit Information Fusion**: After receiving the logit from each client, the server performs the global logit information fusion for this round. Then, the server sends down the global logit to each client.

**(4) Model Training Based on Knowledge Distillation Method**: Each client receives the global logit information and uses the knowledge distillation method to train the local model. Each client treats the global logit information as the teacher model output information and the local model as the student model.

The entire federated task repeats these steps until the specified communication rounds are completed.

#### 3.1.2. The Problem of Heterogeneous Federated Learning

Although some federated knowledge distillation methods solve the heterogeneous model fusion problem, these methods assume that the logit information output by each client is trustworthy. In practical scenarios, a malicious client-side may tamper with the logit information to launch an attack behavior. Therefore, the following three problems are encountered during federated heterogeneous model fusion.

**(1) Client Identification Problem**: Some clients may intentionally upload malicious logit information to launch attacks. If the server uses this malicious information for global information fusion, this will seriously affect the accuracy of global information.

**Figure 2:**
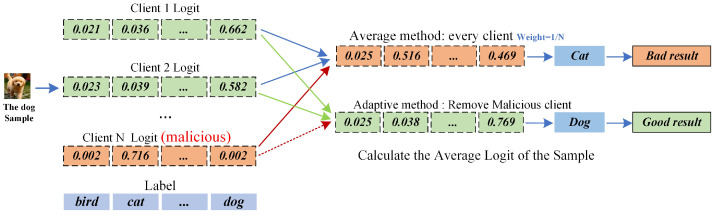
Logit attack behavior of malicious clients.

We use the CIFAR-10 dataset as an example to describe the problem, as shown in Figure 2. The CIFAR-10 is a 10-categorical dataset, and the logit output by each client for the samples in this dataset is a vector of probabilities of length 10, with each element of the vector representing the probability of belonging to a certain category. We assume that there are *N* clients involved in the fusion of global information, and the *N*-th client is malicious. Each client outputs logit information for the same sample data, and the *N*-th client outputs a malicious logit. If the server side directly adopts the average fusion method for information fusion, this will lead to the final prediction result being the cat. However, the actual sample’s category is a dog.

To solve this problem, we must design a client identification method to filter out malicious information to participate in the global information fusion. We assume that n clients participate in the global information fusion. We define $C_{i}$ as the ith client, whereas the set of n clients is defined as $\left\{C_{1},C_{2},\ldots ,C_{n}\right\}$ . Then, the problem is shown in Equation (1). The SelectTrustworthyClient(.) is a method that selects the trustworthy client from all the clients.

(1) $$\left\{C_{1},C_{2},\ldots ,C_{k}\right\}\leftarrow \mathrm{SelectTrustworthyClient}\left(\left\{C_{1},C_{2},\ldots ,C_{n}\right\}\right)$$

**(2) Global Knowledge Fusion Problem**: When the server identifies the malicious client, the next step of the server is to use the selected trustworthy client for global information fusion. Due to the problem of computational resources and data distribution of the clients, this leads to quality differences in the accuracy of the output logit information of each client model, e.g., some clients may output incorrect or low-quality logit. The traditional federated heterogeneous model methods (FedMD, FedDistill) directly use the average weight fusion method to fuse the logit information, and these methods are unreasonable. To solve this problem, we must design an adaptive information fusion method to achieve high-quality global information fusion. We define the logit information output by the ith client as $L_{i}$ , and then the logit set output by all clients is defined as $\left\{L_{1},L_{2},\ldots ,L_{k}\right\}$ . The problem is shown in Equation (2).

(2) $$L_{\mathrm{global}}\leftarrow \mathrm{FusionGlobalLogit}\left(\left\{L_{1},L_{2},\ldots ,L_{k}\right\}\right)$$

**(3) Client Knowledge Distillation Problem**: In each communication round of federated learning, the server sends the global logit information to each client. How to use this global knowledge to train the local model is also a problem. We also use the knowledge distillation approach to solve this problem, where we treat the global logit information as the teacher model and the client’s model as the student model. We use the knowledge distillation method to transfer global information from the teacher to the student model. However, in real scenarios, the information output by the teacher model may also have errors. In that case, this will lead to the client using the wrong knowledge to guide the training of the local model, so we need to design an adaptive knowledge distillation method to solve this problem. This method can select the correct knowledge to guide the student model. We define $M_{i}$ as a model of the ith client, and the set of models of clients is denoted as $\left\{M_{1},M_{2},\ldots ,M_{k}\right\}$ . Then, we define the problem as Equation (3).

(3) $$\left\{M_{1},M_{2},\ldots ,M_{k}\right\}\leftarrow \mathrm{AdaptiveKD}\left(L_{\mathrm{global}}\right)$$

The AdaptiveKD(.) is the adaptive knowledge distillation method, which can adaptively transfer the server-side global knowledge to the client models.

In summary, To accomplish trustworthy federated learning in a heterogeneous environment, we need to solve the following problems.

**(1) Client Identification Problem**: How to identify a malicious client based on logit information. We must exclude malicious clients from global knowledge fusion.

**(2) Global Knowledge Fusion Problem**: How to adaptively select clients based on the logit information to achieve high-quality global knowledge fusion.

**(3) Global Knowledge Migration Problem**: How to transfer global information from the server to the client and use the global knowledge to guide the client model training.

In this paper, we address the problem of federated heterogeneous model fusion by sharing a labeled dataset between the server and each client. The following four sections, Section 3.2, Section 3.3, Section 3.4 and Section 3.5, will detail how to solve these problems.

### 3.2. Malicious Client Identification Method Based on Logit Feature

Client identification stands as a foundational step in realizing trustworthy federated learning. This process involves identifying and excluding malicious clients, thus preventing their logit information from being incorporated into global information fusion. Our approach shares a public dataset between the server and each client to facilitate knowledge fusion within a heterogeneous federated learning framework. The global knowledge is derived by amalgamating the logit information produced by each client based on this public dataset.

**Figure 3:**
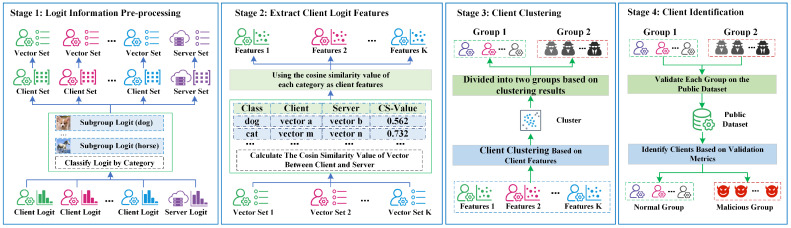
Trustworthy client identification method based on logit feature information.

Drawing on the method of [29], we also train a model at the server and use the model to output each sample’s logit for the public dataset. Since the server-side logit information is independent of the client model, this ensures that the server-side logit information is trustworthy. We can compare the logit information of each client and server to identify the type of client. The process consists of four stages, as shown in Figure 3.

Before describing these stages, we define the following elements. We define the set of clients as $\left\{C_{1},C_{2},\ldots ,C_{k}\right\}$ , and the public dataset is denoted as $D_{0}$ ; it has a total of m samples and n categories. We define $s_{j}$ as the *j*-th sample in the public dataset $D_{0}$ , and $l_{i}^{j}$ as the logit corresponding to the sample $s_{j}$ of client $C_{i}$ , then the set of logits on the public dataset output by the *i*-th client is $\left\{l_{i}^{1},l_{i}^{2},\ldots ,l_{i}^{m}\right\}$ . Similarly, we define the set of logit output from the server side as $\left\{l_{s}^{1},l_{s}^{2},\ldots ,l_{s}^{m}\right\}$ .

**Stage 1 (Preprocessing of Logit Information)**: we first preprocess the logit information from each client and server, which mainly includes two steps: (1) logit information grouping and (2) vectorizing each subgroup logit.

(1) Logit information grouping: When the server receives the logit information uploaded by the client, it first groups the logit information according to its category. The process is shown in Equation (4).

The GroupByCategory(.) denotes grouping sample logit according to the sample category, where $G_{i}^{j}$ is the *j*-th subgroup of the *i*-th client, and each client has n subgroups.

(4) $$\left\{G_{i}^{1},G_{i}^{2},\ldots ,G_{i}^{n}\right\}\leftarrow \mathrm{GroupLogitByCategory}\left(\left\{l_{i}^{1},l_{i}^{2},\ldots ,l_{i}^{m}\right\}\right)$$

At the same time, we also need to group the server-side logit information, and we define the set of the subgroup of the server as $G_{s}=\left\{G_{s}^{1},G_{s}^{2},\ldots ,G_{s}^{n}\right\}$ .

(2) Vectorize sub-grouping logit: After finishing logit grouping, we need to merge logit within each sub-grouping into a one-dimensional vector. We define $v_{i}^{j}$ as *j*-th vector of *j*-th subgroup, and then *n* subgroups correspond to *n* vectors. The process is shown in Equation (5).

(5) $$\left\{v_{i}^{1},v_{i}^{2},\ldots ,v_{i}^{n}\right\}\leftarrow \mathrm{VectorLogit}\left(\left\{G_{i}^{1},G_{i}^{2},\ldots ,G_{i}^{n}\right\}\right)$$

VectorLogit(.) is denoted as a vectorized subgroup. We define the set of vectors for the *i*-th client as $V_{i}$ , where $V_{i}=\left\{v_{i}^{1},v_{i}^{2},\ldots ,v_{i}^{n}\right\}$ .

Repeating steps (1) and (2), we compute the set of vectors for each client and server, respectively.

**Stage 2 (Logit Feature Extraction)**: In the previous phase, we extracted the client and server logit vectors, and in this stage, we need to compute the logit feature values for each client based on the vector information. Since we also output the logit information independently on the server side, this can guarantee that the server-side logit information is trustworthy. Therefore, we can identify the client type by comparing the similarity between the client-side and server-side vectors.

To ensure trustworthy federated learning, we employ cosine similarity as a key metric to compare the server’s and clients’ vectors. Cosine similarity values range from −1 to 1, indicating the degree of similarity between two vectors. A value close to 1 signifies that the vectors are nearly identical, whereas a value approaching −1 indicates that the vectors are opposed. In this context, the eigenvectors of a malicious client are expected to differ significantly from those on the server side. Conversely, a trustworthy client’s vectors will consistently align closely with the server’s. This characteristic variance in cosine similarity values provides a reliable means to identify trustworthy clients.

We assume that there are n categories in the public dataset, and for the *j*-th category, we define the *j*-th vector of the *i*-th client as $v_{i}^{j}$ and the vector of the server as $v_{s}^{j}$ . We compute the client cosine similarity value using Equation (6). Then, we compute the cosine similarity values for all categories on the client side in turn.

(6) $${cs}_{i}^{j}=\mathrm{cosim}\left(v_{i}^{j},v_{s}^{j}\right)=\frac{v_{i}^{j}\cdot v_{s}^{j}}{∥v_{i}^{j}∥\cdot ∥v_{s}^{j}∥}\;,j\in \left[1,n\right]$$

The set of all categories of the *i*-th client is expressed as $\left\{cs_{i}^{1},cs_{i}^{1},\ldots ,cs_{i}^{m}\right\}$ . We take the cosine similarity set of each client as the client’s feature value.

**Stage 3 (Clustering based on client features)**: In this stage, we use the client features extracted in the previous step and group the clients into two clusters using the clustering method, as shown in Equation (7).

(7) $$\left\{{Group}_{1},{Group}_{2}\right\}\leftarrow \mathrm{Cluster}\left(\left\{CS_{1},CS_{2},\ldots ,CS_{k}\right\}\right)$$

represents the clustering method. $G_{1}$ and $G_{2}$ represent two class clusters, respectively.

**Stage 4 (Verify Clients)**: In the previous stage, we divided the clients into two groups. However, we do not know which group is normal, so we need to verify these two groups. We use the accuracy of the clients in each group on the public dataset to determine whether they are credible clients. The group with the highest accuracy is the trusted client group, and the lower group is the malicious client group. The process is shown in Equation (8).

(8) $$\left\{{Acc}_{1},{Acc}_{2}\right\}\leftarrow \mathrm{ValidateGroupAcc}\left(\left\{{Group}_{1},{Group}_{2}\right\}\right)$$

Although this step can identify most malicious clients, some malicious clients (e.g., noise attack clients) can escape the method. To further exclude malicious clients, we need to verify the accuracy of the clients in the normal group. We first validate the accuracy of each client on the public dataset and then calculate the average accuracy of those clients. Finally, we calculate the difference between the client and average accuracy. We also exclude clients from participating in global logit calculations when their accuracy is below the average value and the difference exceeds a set threshold. The process is shown in Algorithm 1.

### 3.3. Global Information Fusion Methods

**Figure 4:**
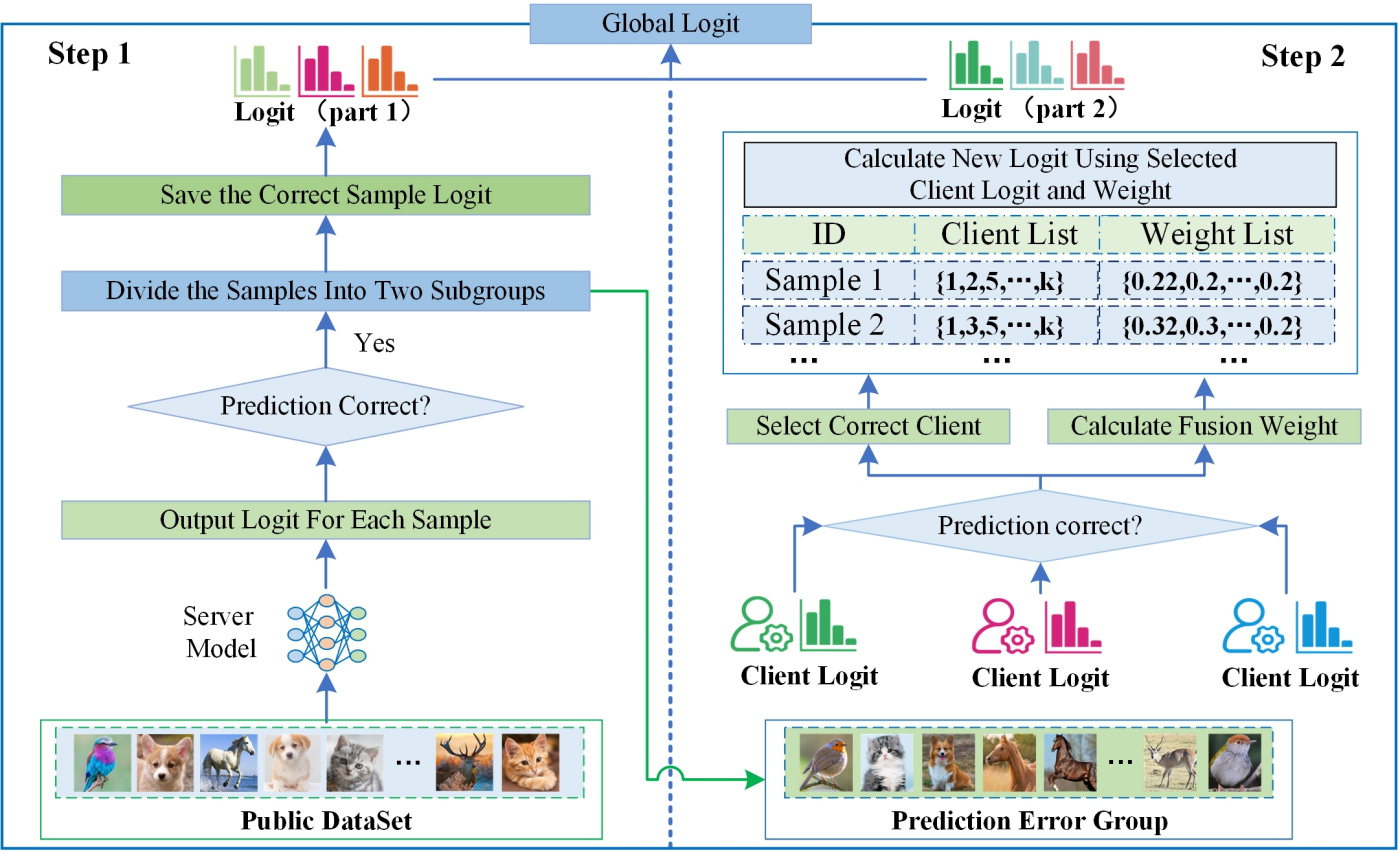
The calculation process of global logit.

Once we have identified the malicious clients, our next task is to use these trustworthy clients’ logit to fuse the global logit. Most federated knowledge distillation methods (such as FedMD and FedDF) use average weight methods to fuse the global logit, which calculates the global logit value by the average of all clients’ logit value, and each client is given the same weight. However, in real scenarios, each client’s data distribution and computational resources are different, which leads to differences in the quality of the logit for the same sample. At the same time, some clients may output an error or a low-quality logit. Using the error logit to fuse the global logit will affect the accuracy of the global information. Therefore, we need to filter out the error information to improve the performance of global information. To accomplish this goal, we design a trustworthy logit calculation method that calculates the global logit in two parts. The flow of the method is shown in Figure 4. The flow of the algorithm is shown in Algorithm 2.
**Algorithm 1** Client identification algorithms

**Input:** 
Each client’s Logit: $L=\{L_{1},L_{2},\cdots ,L_{n}\}$ , Public Data: $D_{0}$

**Output:** 
Trustworthy Clients $C=\{C_{1},C_{2},\cdots ,C_{K}\}$
1:/* Center Node Process */2:**for** Round *t* from 1 to *T* **do**3:    Init Trustworthy Client List TC_List={} // Initialise client features List4:    
$M_{s}\leftarrow \mathrm{TrainServerModel}\left(D_{0}\right)$
5:    
$\left\{l_{s}^{1},l_{s}^{2},\cdots ,l_{s}^{m}\right\}\leftarrow \mathrm{OutPutLogit}\left(M_{s},D_{0}\right)$ // Output logit on the Public Dataset6:    
$\left\{G_{s}^{1},G_{s}^{2},\cdots ,G_{s}^{n}\right\}\leftarrow \mathrm{GroupLogitByCategories}\left(\left\{l_{s}^{1},l_{s}^{2},\cdots ,l_{s}^{m}\right\}\right)$
7:    
$\left\{v_{s}^{1},v_{s}^{2},\cdots ,v_{s}^{n}\right\}\leftarrow \mathrm{VectorLogitEachCategories}\left(\left\{G_{s}^{1},G_{s}^{2},\cdots ,G_{s}^{n}\right\}\right)$
8:    **for** Client $C_{i}$ from 1 to *K* **do**9:         
$\left\{G_{i}^{1},G_{i}^{2},\cdots ,G_{i}^{n}\right\}\leftarrow \mathrm{GroupLogitByCategories}\left(\left\{l_{i}^{1},l_{i}^{2},\cdots ,l_{i}^{n}\right\}\right)$
10:        
$\left\{v_{i}^{1},v_{i}^{2},\cdots ,v_{i}^{n}\right\}\leftarrow \mathrm{VectorLogitEachCategories}\left(\left\{G_{i}^{1},G_{i}^{2},\cdots ,G_{i}^{n}\right\}\right)$
11:        
$\left\{cs_{i}^{1},cs_{i}^{2},\cdots ,cs_{i}^{n}\right\}\leftarrow \mathrm{CalculateCosineValue}\left(\left\{G_{i}^{1},G_{i}^{2},\cdots ,G_{i}^{n}\right\}\right)$
12:        
$CS_{i}\leftarrow \mathrm{GetClientFeature}\left(\left\{cs_{i}^{1},cs_{i}^{2},\cdots ,cs_{i}^{n}\right\}\right)$
13:    
$\left\{{\mathrm{Group}}_{1},{\mathrm{Group}}_{2}\right\}\leftarrow \mathrm{Cluster}\left(\left\{CS_{i},CS_{2},\cdots ,CS_{k}\right\}\right)$ // Clustering the clients14:    
$\left\{Acc_{1},Acc_{2}\right\}\leftarrow \mathrm{ValidateACC}\left(Group_{1},Group_{2}\right)$ // Validation accuracy15:    
$UC\_List\leftarrow \mathrm{AddMaxAccGroup}(\left\{C_{a},C_{b},\cdots ,C_{k}\right\})$
16:    
$A_{avg}\leftarrow \mathrm{CalculateAvgAcc}\left(\left\{A_{1},A_{2},\cdots ,A_{k}\right\}\right)$
17:    **for** client $C_{j}$ from 1 to *t* in UC_List **do**18:        **if** $\left|A_{i}-A_{avg}\right|>\epsilon$ **then**19:           
$TC\_List\leftarrow \mathrm{AddClientToList}(A_{i})$
20:Output Trustworthy Clients $TC=\{C_{a},C_{b},\cdots ,C_{K}\}$



In our method, we train a model on the server side using the public dataset, and we use the server model to output the logit of each sample of the public dataset. After we obtain all the samples’ logit, we divide the logit into two groups according to whether the predicted result of the logit is consistent with the actual labels of the sample. We divide the logit into the correctly predicted and the incorrectly predicted groups. When the server model has incorrect predictions, it means that the server side cannot predict this information, which requires the integration of logit from the clients to obtain the correct value [29]. We calculate the logit in two parts according to the grouping of the samples, and the calculation process is shown in Figure 4.

We define $\left(x_{i},y_{i}\right)$ as the *i*-th sample and the label, and the $D_{0}$ as the public dataset. Suppose $D_{0}$ is a K-categorical dataset with m samples. The set of all samples is denoted as $D_{0}=\left\{\left(x_{1},y_{1}\right),\left(x_{2},y_{2}\right),\ldots ,\left(x_{n},y_{n}\right)\right\}$ , $y_{i}\in \left[1,K\right]$ . We define $l_{i}^{m}$ as the *m*-th sample logit output by the *i*-th client and define $L_{i}$ as the set of the logit of the public dataset output by client $C_{i}$ . The set is denoted as $L_{i}=\left\{l_{i}^{1},l_{i}^{2},\ldots ,l_{i}^{m}\right\}$ .

We define $l_{s}^{m}$ as the *m*-th sample’s logit output by the server model and $p_{s}\left(x_{m}\right)$ as the predicted label of the server model for the sample $x_{m}$ . Then, we calculate the global logit for the sample $x_{m}$ based on the results of the server-side prediction. We define $l_{global}^{m}$ as the global logit of the *m*-th sample. The $\mathrm{FusionLogit}(\left\{l_{1}^{m},l_{2}^{m},\cdots ,L_{k}^{m}\right\})$ represents fusing k clients’ logit to calculate the new global logit for the sample $x_{m}$ . We use Equation (9) to calculate all the sample logit in turn. We will introduce how to calculate the logit in the following.

(9) $$l_{global}^{m}=\left\{\begin{array}{cc} l_{s}^{m} & ,p_{s}\left(x_{m}\right)=y.\\ \mathrm{FusionLogit}(\left\{l_{m}^{1},l_{m}^{2},\cdots ,l_{m}^{k}\right\}) & ,p_{s}\left(x_{m}\right)\neq y. \end{array}\right.$$

**Part 1**: If the server-side output logit is consistent with the actual category of labels, we directly use the server-side model output logit as part of the global logit.

**Part 2**: If the server-side output logit is wrong, we will obtain the logit from the client’s logit. In this condition, we can regard the global logit calculation problem as a multi-teacher logit fusion problem. Each client’s logit is the output of a teacher model, and our goal is to obtain the global logit from those teachers’ logit. However, the quality of logit output from each teacher model is different. To adaptively fuse multiple teachers’ logit information, we need to assign higher weights to high-quality information while assigning lower weights to low-quality information. We draw on the paper’s method [30] to calculate the weight value. This calculation process consists of three steps.

**(1) The Calculation of Fusion Weight**: We first calculate the cross-entropy loss for each client, where k denotes the *k*-th client. This loss can reflect the confidence level of each client in the sample [30]. The calculation process is shown in Equation (10).

(10) $$L_{CE}^{k}=-\sum_{1}^{C}y^{c}\log\left(Softmax\left(L_{c}^{k},T\right)\right)$$

where $Softmax(L_{c}^{k},T)$ denotes the calculation of $L_{c}^{k}$ at temperature T using the softmax function. Then, we calculate the weight of each client according to Equation (11).

(11) $$w^{k}=\frac{1}{K-1}\left(1-\frac{\exp(L_{CE}^{k})}{\sum_{1}^{j}\exp L_{CE}^{j}}\right)$$

Unlike [30], we calculate each client’s weight for each category according to logit categories. We first group each client’s logit categories on the public dataset’s output and then use Equation (11) to calculate the client’s fusion weight. Eventually, we obtain a weight matrix where each row represents each category of logit, and each column represents each client’s weight. The weight matrix is shown in Equation (12).

(12) $$W\left(C,Y\right)=\left[\begin{array}{cccc} w_{11} & w_{12} & \cdots & w_{1k}\\ \cdots & \cdots & \cdots & \cdots \\ w_{m1} & w_{m2} & \cdots & w_{mk} \end{array}\right]$$

**(2) Re-adjust Client Weight**: After determining each client’s weight for each category, it is essential to address the issue of incorrect logit outputs. Some clients may produce an error logit for a sample, which necessitates the removal of these incorrect logits from the fusion process. When computing the logit for a sample, if an incorrect logit is identified and removed, the fusion weight assigned to that logit should consequently be reduced to zero. This adjustment then requires recalibrating the fusion weights for the remaining clients to maintain the balance in the model’s overall learning. To recalibrate these weights, we employ L1-normalization, which effectively redistributes the weights among the remaining clients. The $p_{k}\left(x\right)$ is the *k*-th client prediction, and y is the label of a sample *x*. The calculation process of the new weight is shown in Equation (13).

(13) $$w^{k}=\left\{\begin{array}{cc} 0 & ,p_{k}\left(x\right)\neq y.\\ \frac{w_{j}}{\sum_{j=1}^{N}\left|w_{j}\right|} & ,p_{k}\left(x\right)=y. \end{array}\right.$$

where $\left|w_{j}\right|$ is the L1-normalization for all the correct logits.

**(3) New Logit Calculation**: Having obtained the revised weights in the previous stage, we now proceed to recalculate the logit for each sample using these new weights in conjunction with the logit information from each client. The process for this new logit calculation is outlined in Equation (14).

(14) $$L_{\mathrm{new}\;}=\sum_{1}^{K}w^{k}L_{n}^{k}$$

When we obtain the logit of all the samples in part 1 and part 2, we merge these two to form the global logit for this communication round. Then, the server sends this global logit down to each client. The process is shown in Algorithm 2.
**Algorithm 2** The Calculation of Glogit Logit Information

**Input:** 
Client Logit List: $L=\{L_{1},L_{2},\cdots ,L_{k}\}$ . Public Dataset $D_{0}$

**Output:** 
Global Logit $L_{g}=\left\{l_{1},l_{2},\cdots ,l_{m}\right\}$
1:/*Public Dataset Logit Calculate Process*/2:CrroctList = {} // Initialise IDs of Correctly predicted sample Logit3:ErrorCrroctList = {} // Initialise IDs of Error predicted sample Logit4:NewCrroctList = {} // Initialise IDs of new calculate sample Logit5:
$M_{s}\leftarrow \mathrm{TrainServerModel}\left(D_{0}\right)$
6:
$\left\{l_{1},l_{2},\cdots ,l_{m}\right\}\leftarrow \mathrm{OutPutLogit}\left(M_{s},D_{0}\right)$ // OutPut Logit on the Public Dataset7:
$W(C,Y)\leftarrow \mathrm{CalculateWeightMatirc}(\left\{L^{1},L^{2},\cdots ,L^{k}\right\})$
8:/**The Calculation of Logit in Part-1**/9:**for** Logit $l_{s}$ from 1 to *m* in Public Dataset **do**10:    **if** $l_{s}=label$ **then**11:        
$CrroctList\leftarrow \mathrm{AddLogit}(l_{s})$
12:    **else**13:        
$ErrorList\leftarrow \mathrm{AddLogit}(l_{s})$
14:/**The Calculation of Logit in Part-2**/15:**for** Logit $l_{s}$ from 1 to *n* in ErrorList **do**16:    Init $SumLogit_{s}=0$ // Initialise the Logit value for this sample17:    **for** Client $C_{i}$ from 1 to $C_{k}$ **do**18:        
$l_{s}\leftarrow \mathrm{GetLogitFromClient}\left(C_{i}\right)$
19:        
$w_{s}\leftarrow \mathrm{GetWeightFromWeightMatric}\left(C_{i}\right)$
20:        
$SumLogit_{s}=SumLogit_{s}+w_{s}\cdot l_{s}$
21:    
$l_{s}=SumLogit_{s}$
22:    
$NewCorectList\leftarrow \mathrm{AddNewLogit}(l_{s})$
23:
$L_{g}=\mathrm{MergeTwoPartLogit}\left(\left\{CrroctList,NewList\right\}\right)$
24:Output Global logit $L_{g}$



### 3.4. Calculation of Weighting Parameters Based on Teacher Logit Confidence Level

Having calculated the global logit information, the server will distribute the global logit to each client. In this phase, clients use the global logit as the output of a teacher model, training their local models on the public dataset via knowledge distillation. Traditional knowledge distillation involves a combined loss function, consisting of cross-entropy loss ( $L_{CE}$ ) and distillation loss ( $L_{KD}$ ), with a balance weight α . The total loss ( $L_{total}$ ) used to optimize the model is shown in Equation (15):

(15) $$L_{total}=\alpha L_{CE}+\left(1-\alpha \right)L_{KD}$$

The values of α are fixed in the traditional knowledge distillation method throughout the model training process. However, in the heterogeneous federated learning knowledge fusion process, the data distribution and computational resources are different across clients, leading to quality differences in logit. When we use these client logits to compute the global logit, the accuracy of the global logit is dynamically changing, which will make the accuracy of the global logit different for each category in the same communication round [31]. To address this, an adaptive knowledge distillation method is required, one that can adjust weight parameters in line with sample categories to enhance client model accuracy. Thus, we propose a redefined equation for $L_{total}$ , as shown in Equation (16), where $w_{k}$ represents the weight for the *k*-th category.

(16) $$L_{total}=\left(1-w_{k}\right)L_{CE}+w_{k}L_{KD}$$

**Figure 5:**
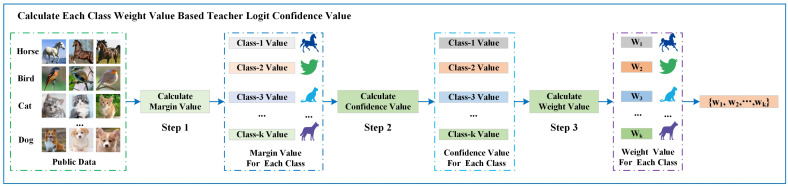
Calculation of weighting parameters based on teacher logit confidence level.

Next, we calculate each category’s $w_{k}$ value. The value is determined by the accuracy of the global logit on the public dataset, which means that the higher the accuracy of the global logit for a category, the higher the global logit confidence value in that category. If the global logit has a low accuracy for one category, this means that the global logit is not credible in that category. To prevent the global logit’s incorrect knowledge from passing to the client model, we need to set $w_{k}$ to 0. Therefore, we can calculate the $w_{k}$ by evaluating the confidence value of the global logit. We borrow the calculation method from [32]. We divide the weight calculation process into three steps, and this process is shown in Figure 5.

**Step 1 (Marginal Value Calculation)**: We need first to calculate the marginal value of each category, which can measure the confidence value of the teacher model in each category in the public dataset, and the larger the marginal value, the higher the prediction accuracy of the teacher model for that category. We define $M_{k}\left(k,p^{t}\left(x\right)\right)$ as the marginal value of the sample *x*. The $p^{t}\left(x\right)$ is the probability that the teacher model predicts a sample *x*, where k is the actual category of the sample, and $k^{\prime }$ is the other category. The *L* is the total number of categories. We obtain the marginal value by calculating the difference between the probability of predicting the sample as category *k* and the probability of predicting the sample as any other category. The calculation process is shown in Equation (17).

(17) $$M_{k}\left(k,p^{t}\left(x\right)\right)=p_{k}^{t}\left(x\right)-\frac{1}{L-1}\sum_{k^{\prime }\neq k}p_{k^{\prime }}^{t}\left(x\right)$$

**Step 2 (Confidence Value Calculation)**: After calculating each sample’s marginal value, we first sum up the marginal values of each category, and then we calculate the average value of the marginal values of each category. We define $C_{value}^{k}$ as the confidence value of the *k*-th category. The calculation process of $C_{value}^{k}$ is shown in Equation (18).

(18) $$C_{value}^{k}=E_{x|k}\left[M_{k}\left(k,p_{k}^{t}\left(x\right)\right)\right]$$

**Step 3 (Weight Calculation)**: When we calculate the value of the $C_{value}^{k}$ , we can get the value of the $w_{k}$ based on this value. Notably, as the accuracy of global logit typically increases over communication rounds, the confidence value of the global logit for each category will be close to 1 after a certain number of rounds. At this point, if we directly use this value ( $C_{value}^{k}=1$ ) to determine the weight value ( $w_{k}=1$ ), it will make the weight value of $L_{CE}$ become 0 ( $1-w_{k}=0$ ), leading to ignoring the loss of the actual label of the sample. However, we aim to use the two losses fully to complete the model training. Therefore, we added the parameter β to limit the value of $C_{value}^{k}$ , which prevents the value from being too large and leads to the problem of ignoring the $L_{CE}$ during the training process. The process is shown in Equation (19).

(19) $$w_{k}=\left\{\begin{array}{cc} 0 & ,C_{value}^{k}\leq 0.\\ \left(1-\beta \right)\cdot C_{value}^{k} & ,C_{value}^{k}>0. \end{array}\right.$$

When $C_{value}^{k}\leq 0$ , it indicates that the global logit has low confidence in the category *k*. We directly ignore the teacher’s knowledge in this situation. When $C_{value}^{k}>0$ , it indicates that the global logit has higher confidence in the class. We set the value of β according to different data distribution scenarios, and we will describe the setting of this value in the subsequent experimental section.

### 3.5. Client Model Training Based on Adaptive Knowledge Distillation

**Figure 6:**
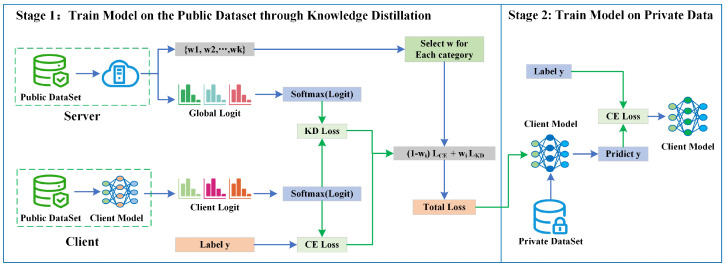
The process of client model training based on adaptive knowledge distillation.

This phase completes the client model training, which consists of two steps: (1) training the client model on the public dataset using adaptive knowledge distillation and (2) model retraining on the private dataset. The process is shown in Figure 6, and the algorithm flow is shown in Algorithm 3.

**(1) Train Model on Public Dataset by Adaptive Knowledge Distillation**: We regard the global logit as the output of the teacher model and the client model as the student model. Then, we use the knowledge distillation method to complete the training of the client model. We define the predicted value of the teacher’s model for the sample $x_{i}$ as $p^{t}\left(x_{i}\right)$ . We define $l_{k}$ as the logit of a sample $x_{i}$ . We calculate the predicted value at sample temperature *T* by the softmax function. The calculation is shown in Equation (20).

(20) $$p_{k}^{t}\left(x\right)=\frac{e^{l_{k}\left(x\right)/T}}{\sum_{j=1}^{K}e^{l_{j}\left(x\right)/T}}$$

We define $L_{CE}$ as the cross-entropy loss of the client. We can calculate the cross-entropy loss by client model prediction with the label y, as shown in Equation (21).

(21) $$L_{CE}=\frac{1}{N}\sum_{n=1}^{N}\sum_{k=1}^{K}-y_{k}\log\left(p_{k}\left(x\right)\right)$$

We define the knowledge distillation loss as $L_{KD}$ . The knowledge distillation loss $L_{K}D$ is obtained by calculating the KL loss between the teacher and student models. The calculation is shown in Equation (22).

(22) $$L_{KD}=\frac{1}{N}\sum_{n=1}^{N}\sum_{k=1}^{K}-p_{k}^{t}\left(x_{i}\right)\log\left(p_{k}\left(x\right)\right)$$

Before each communication round starts, the server sends the global logit and weight list to each client, and the client selects the $w_{k}$ adaptively according to the sample category. We define the set of weights for communication round t as $\left\{w_{1},w_{2},\ldots ,w_{k}\right\}$ . Each client completes the computation of the loss of each category according to the category weights $w_{k}$ . The calculation process is shown in Equation (23).

(23) $$L_{total}=\left(1-w_{k}\right)L_{CE}+w_{k}L_{KD}$$

Finally, each client uses the public dataset and the adaptive knowledge distillation method to train the local model.

**(2) Model training using Private Dataset**: In this stage, clients continue to train their local models on their private datasets using the model obtained from the previous phase. We use cross-entropy loss during model training at this node to optimize the model. We define the cross-entropy loss as $L_{private\_CE}$ and the client model prediction as $p_{k}\left(x\right)$ , where *x* is the private sample of the client, and *k* is the sample category. Then, we obtain $L_{private\_CE}$ by calculating the model prediction with the actual labels of *y*. The process is shown in Equation (24).

(24) $$L_{private\_CE}=\frac{1}{N}\sum_{n=1}^{N}\sum_{k=1}^{K}-y_{k}\log\left(p_{k}\left(x\right)\right)$$

**Algorithm 3** Train Client Model
**Input:** Global Logit: $L_{g}$ , Each categories weight: $W=\{w_{1},w_{2}\ldots w_{k}\}$

**Output:** 
Trained Client Model $M=\{M_{1},M_{2}\ldots M_{n}\}$
1:/* Each Client Training Step */2:**for** $C_{i}$ from 1 to $C_{n}$ **do**3:    /* **Step 1:Train Client Model on Public Dataset** */4:    
$M_{0}\leftarrow \mathrm{InitClientModel}\left(round\right)$ // Initiate the model with the previous model5:    
$pre\leftarrow \mathrm{PredValueOnPublicData}(M_{0})$
6:    
$L_{CE}\leftarrow \mathrm{GetCELoss}\left(y_{pre},y_{label}\right)$ //Calculate CE Loss on Public Dataset7:    
$L_{KD}\leftarrow \mathrm{GetKDLoss}\left(\{softmax\left(y_{pre}\right)\right\},L_{g})$ //Calculate KD Loss on Public Dataset8:    
$w_{k}\leftarrow \mathrm{SelectKDWeightByCategories}\left(\left\{w_{1},w_{2}\ldots w_{k}\right\}\right)$
9:    
$L_{total}=\left(1-w_{k}\right)*L_{CE}+w_{k}*L_{KD}$
10:    
$M_{i}\leftarrow \mathrm{TrainClientModel}\left(L_{total}\right)$
11:    /* **Step 2:Train Client Model on Private Dataset** */12:    
$y_{pre}\leftarrow \mathrm{PredValueOnPrivateData}\left(M_{i}\right)$
13:    
$L_{prviate\_CE}\leftarrow \mathrm{GetCELoss}\left(y_{pre},y_{label}\right)$ //Calculate CE Loss on Private Dataset14:    
$M_{i}^{\prime }\leftarrow \mathrm{TrainClientModel}\left(L_{prviate\_CE}\right)$
15:Output Model of Clients = $\left\{M_{1}^{\prime },M_{2}^{\prime },\ldots ,M_{n}^{\prime }\right\}$



## 4. System Design

**Figure 7:**
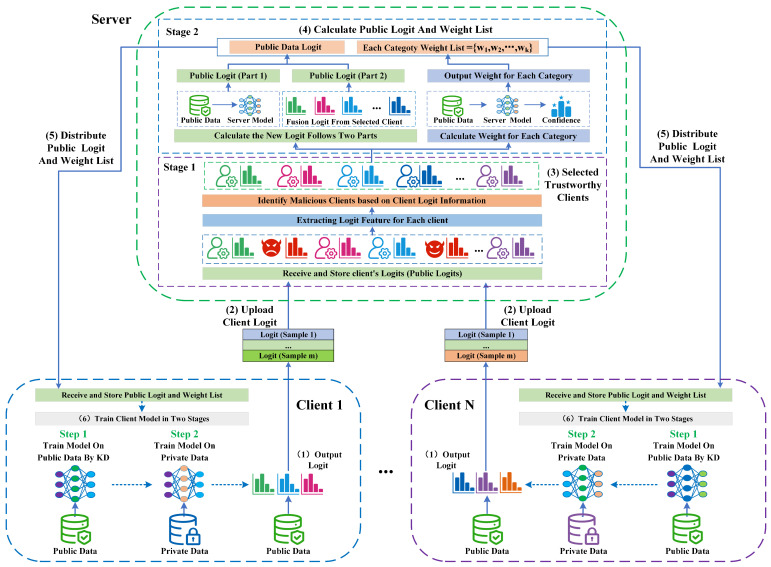
The system architecture of FedTKD.

In addressing the challenges of trustworthy federated learning in heterogeneous environments, we design the FedTKD framework. This framework includes two core processes: client model training through adaptive knowledge distillation and trustworthy information fusion on the server side. The server is responsible for client identification and the fusion of global information, while the client focuses on model training and logit information output. The process is shown in Figure 7, and the algorithm flow detailed in Algorithm 4.

**(1) Initialization Phase**: The server distributes a public dataset to each client. This dataset is sent only once at the beginning.

**(2) Output Client Logit**: Clients train their local models using both public and private datasets. Then, each client outputs a logit for each sample in the public dataset using the local model.

**(3) Upload Logit Information**: After generating logits for the public dataset, clients upload this logit information to the server. We have designed an efficient logit information storage format, as shown in Equation (25), where each sample in the public dataset corresponds to a unique piece of information.

(25) {Index,Logit[class_1_value,class_2_value,…,class_n_value]}

**(4) Global Logit Information Fusion**: Upon receiving logit from all clients in a communication round, the server identifies the type of each client and selects the information from trustworthy clients to perform global logit fusion. This is a three-step process:

Step 1 (Identify Client): The server uses Algorithm 1 to distinguish between malicious and trustworthy clients.

Step 2 (Trustworthy Logit Fusion): The server calculates new global logit information using Algorithm 2.

Step 3 (Category Weight Calculation): After global logit calculation, the server computes the weight value for each category based on confidence levels.

**(5) Distribute Global Logit and Weight List**: The server sends the latest global logit information and category weight list to each client for the new round.

**(6) Train Client Model**: This stage is crucial for transferring knowledge from the global knowledge base to each client model. It includes two steps.

Step 1 (Model Training Based on Adaptive Knowledge Distillation): Clients receive the global logit and weight list and use this information to train the local model on the public dataset.

Step 2 (Client Model Retraining): The client uses the model from the previous phase and continues to train the local model on the private dataset.
**Algorithm 4** The Workflow of FedTKD

**Input:** 
Client Model: $M=\{M_{1},M_{2},\ldots M_{n}\}$ , Private Dataset: $D=\{D_{1},D_{2},\ldots D_{n}\}$ , Public Dataset: $D_{0}$ , Communication round: T.
**Output:** 
Trained client model $M=\{M_{1}^{\prime },M_{2}^{\prime },\ldots ,M_{n}^{\prime }\}$
1:/* Client Process */2:**for** round *t* from 1 to *T* **do**3:    **for** $C_{k}$ from 1 to $C_{n}$ **do**4:        /* **Step 1: Train Client Model On Public Data** */5:        
$M_{k}\leftarrow \mathrm{InitClientModel}\left(t\right)$ // Load Previous Model6:        
$L_{g}^{t}\leftarrow \mathrm{RecieveGlobalLogit}\left(L_{g}^{t}\right)$
7:        
$M_{k}^{1}\leftarrow \mathrm{TrainModelByAKD}\left(D_{0},M_{k}^{1},L_{g}^{t}\right)$ // According to Algorithm 38:        /* **Step 2: Train Client Model On Private Data** */9:        
$M_{k}^{2}\leftarrow \mathrm{TrainLocalModel}\left(D_{k},M_{k}\right)$
10:        /* **Step 3: Output Logit On Public Dataset** */11:        
$\left\{l_{k}^{1},l_{k}^{2},\cdots ,l_{k}^{m}\right\}\leftarrow \mathrm{OutPutLogit}\left(M_{k}^{2},D_{0}\right)$
12:        
$Server\leftarrow \mathrm{SendLogitToServer}(L_{k})$
13:/* Server Process */14:**for** round *t* from 1 to *T* **do**15:    **for** $C_{i}$ from 1 to $C_{n}$ **do**16:        
$\left\{L_{1},L_{2},\cdots ,L_{n}\right\}\leftarrow \mathrm{ReceiveClientLogit}\left(\left\{C_{1},C_{2},\cdots ,C_{n}\right\}\right)$
17:    
$\left\{C_{1},C_{2}\ldots C_{k}\right\}\leftarrow \mathrm{IdentifyClientType}\left(\left\{L_{1},L_{2},\cdots ,L_{n}\right\}\right)$ // Algorithm 118:    
$L_{g}^{t}\leftarrow \mathrm{CalculateGlobalLogit}\left(\left\{L_{1},L_{2},\cdots ,L_{k}\right\}\right)$ // Algorithm 219:    
$\left\{w_{1},w_{2},\cdots ,w_{k}\right\}\leftarrow \mathrm{CalculateCategoryWeight}\left(L_{g}^{t}\right)$
20:    
$\left\{C_{1},C_{2},\cdots ,C_{k}\right\}\leftarrow \mathrm{SendLogitAndWeight}\left(L_{g}^{t},W\right)$ //Send to Clients21:    Train Client Model According to global logit $L_{g}^{t}$ and weight list *W*.22:Output Client Model List: $M=\{M_{1}^{\prime },M_{2}^{\prime },\ldots ,M_{n}^{\prime }\}$



## 5. Experiment

### 5.1. Experiment Setup

#### 5.1.1. Experiment Datasets

**Dataset Description**: Our experiment utilizes three categorical datasets: MNIST, Fashion-MNIST, and CIFAR-10, each dataset is described as follows:

MNIST: A dataset for handwritten digit classification consisting of 60,000 training and 10,000 test samples. Each sample is a 28 × 28 grayscale image representing digits from 0 to 9.

Fashion-MNIST: It is a ten-category dataset. This dataset comprises 60,000 training and 10,000 test images, each a single-channel grayscale image.

CIFAR-10: A more diverse dataset with ten categories containing 50,000 training and 10,000 test samples. Each sample is a 32 × 32 color image.

**Figure 8:**
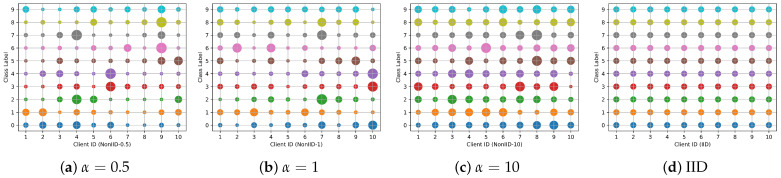
Different data distribution scenarios for the Cifar-10 dataset (10 clients).

**Dataset Preprocessing**: Our algorithm necessitates the construction of a shared public dataset between the server and client sides, alongside private datasets for each client. We achieve this goal through the following preprocessing steps:

(1) Public Dataset Part: A portion of the original dataset is reserved as the public dataset. We ensure a uniform representation by selecting 10% of data from each category. For instance, in CIFAR-10’s ten categories, 500 samples per category are allocated to the public dataset.

(2) Private Dataset Part: To mimic the Non-IID data distribution of each client, We control client data distribution by adjusting the Dirichlet function’s parameter α . A lower α value leads to a more Non-IID distribution, while a higher value approximates an IID (independently identically distributed) scenario. This distribution is visualized in Figure 8, where each dot’s size represents the sample count, and its color indicates the sample category.

#### 5.1.2. Baseline

We compared our method with five baseline algorithms. These include three methods (FedMD, FedDF, and FedDistill), which require sharing public datasets between the server and clients, and two methods (FedDistill+ and FedHe), which do not require public datasets.

**FedMD** [7]: This method solves the heterogeneous model knowledge fusion problem by constructing a public dataset. The server fuses the logit information of each client with the average weight method, and the client uses the knowledge distillation method to train the client model on the public dataset and then migrates the client model to the private dataset for further training.

**FedDF** [8]: This method needs to construct an auxiliary dataset, and the server side integrates the logit information of each client to the global logit. Each client treats the global logit as the teacher’s model knowledge to guide the local model training.

**FedDistill** [9]: This method needs to build a public dataset. The approach applies knowledge distillation methods to federated learning, which uses global logit as a teacher to guide the local model training of each client.

**FedDistill+** [18]: This method does not require a public dataset. Each client first calculates the average logit of each category, and the server side aggregates the logit of each category and calculates the average value. Finally, each client uses the average logit as a regularization loss to guide the local model training.

**FedHe** [33]: This method does not require a public dataset. It also uses the average logit information of each category as a loss to assist client model training.

#### 5.1.3. Metrics

We evaluate the performance of each algorithm by three key metrics, described below.

**(1) Average Client Model Accuracy on the Test Dataset**: This metric assesses the algorithm’s effectiveness in enhancing the accuracy of the client’s model. Higher average accuracy indicates better performance across all clients. We assume that *K* clients participate in federated learning with *m* communication rounds. We denote the accuracy of the *i*-th client in the *t*-th communication round as $A_{t}^{i}$ . Then, we define $A_{avg}^{t}$ as the average client model accuracy for the *t*-th round, and we can calculate the average accuracy of *K* clients for *t*-th rounds according to Equation (26).

(26) $$A_{avg}^{t}=\frac{1}{K}\sum_{i=1}^{K}A_{i}^{t},t\in \left[1,m\right]$$

Then, we define $A_{avg}$ as the set for all communication rounds, and the set is defined as $A_{avg}=\left\{A_{avg}^{1},A_{avg}^{2},\cdots ,A_{avg}^{m}\right\}$ .

**(2) Accuracy of Global Logit on the Public Dataset**: This metric reflects the server’s capability to amalgamate knowledge from each client. When the server calculates the global logit at *t*-th communication rounds, we can test the global logit’s accuracy on the public dataset. We define $A_{global}^{t}$ as the accuracy of global logit. The m communication round set is as shown in Equation (27).

(27) $$A_{gobal}=\left\{A_{gobal}^{1},A_{gobal}^{2},\ldots ,A_{gobal}^{m}\right\}$$

**(3) Accuracy of Client Logit on the Public Dataset**: When clients finish training the local model, each client outputs the logit of the public dataset. We get this metric by calculating the accuracy of the client’s logit on the public dataset. We define $A_{i}^{t}$ as the accuracy of the *i*-th client’s logit on the public dataset at the *t*-th round. Then, the set of all the clients at the *t*-th round is shown in Equation (28).

(28) $$A^{t}=\left\{A_{1}^{t},A_{2}^{t},\ldots ,A_{k}^{t}\right\}$$

#### 5.1.4. Heterogeneous Network Setup

To simulate a heterogeneous federated learning environment, we employed CNN networks as our foundational architecture, customizing them to have varying structures. We categorized the clients into five distinct groups, with each group utilizing a different network structure but maintaining consistency within the group.

**Client Network Setup**: For the MNIST and Fashion-MNIST datasets, we designed five pairs of CNN networks for processing single-channel images. For the CIFAR-10 dataset, we designed five CNN networks processing three channels.

**Server Network Setup**: It is crucial that the parameter of the server model is more complex than that of the client-side model. This ensures the server’s model can fully integrate knowledge from each client model.

We designed experiments with different numbers of clients (10, 15, and 20, respectively) participating in the federation task, and the client network configurations in each experiment are shown in Table 1. Taking the CIFAR-10 dataset as an example, we elaborate on the parameter settings of both client- and server-side network models. Each network is characterized by different convolutional layers and structures. For instance, Conv-X denotes the convolutional module, and C2d(a,b) means the 2D convolution. FC is the fully connected module, and Line(a,b) denotes the linear layer.

#### 5.1.5. Hardware and Software Environment

**Hardware Setup**: Our experiments were conducted on a high-performance workstation equipped with an Intel i9-12900K CPU, 64GB of RAM, and an NVIDIA RTX 3090 graphics card.

**Software Setup**: We developed a federated learning framework (FedBolt) to implement key functionalities such as partitioning client datasets, customizing client training models, simulating various client attacks, and facilitating multi-client co-training.

### 5.2. Experimental Results and Analysis

Our experiments are structured into four parts to validate the performance and reliability of our algorithm: malicious client identification, attack experiments, different data distribution experiments, and framework performance analysis. Notably, the malicious client attack experiments are crucial in evaluating our algorithm’s robustness. We also compare our algorithm against other baselines in both attack and normal scenarios.

#### 5.2.1. Malicious Client Attack Experiment

To verify the performance of each algorithm under different attack scenarios, we designed three attack-type scenarios. The details of each attack scenario are as follows.

**Label Flipping Attack (Type-1)**: In the classification task, each sample’s logit information is a vector of K values, and the maximum of these values is the classification result of the model. We select this maximum value and swap it to a random location. We follow this strategy to replace 50% of the number of samples’ logit to form a label-flipping attack.

**Noisy Data Attack (Type-2)**: We add a certain percentage of noisy data (e.g., Gaussian noise) to the client’s private dataset to train the local model, reducing the client’s model’s accuracy. Then, we use the low-quality model to output the logit in the public dataset, which generates inaccurate logits of the public dataset.

**SecondMax Attack (Type-3)**: We tamper with the logit information corresponding to the sample. We first find the position of the largest value in the logit, then randomly select 50% of the remaining positions, and modify the value of these positions to (MaxValue − 0.00001 ). For example, in a ten-classification task, the element at position 9 is the maximum value ( 3.56789 ). We modify the values of the elements at positions 0, 1, 5, 6, and 8 to ( 3.56789−0.00001 ).

**Federated Task Setup**: Our experiments involved scenarios with 10, 15, and 20 clients participating in a federated task with 100 communication rounds. At the same time, each client performs one epoch of local model training. We divide the clients into two groups: malicious clients and normal clients. Client IDs 1, 3, 5, 7, 9, 11, 13, 15, 17, and 19 are normal clients, and client IDs 2, 4, 6, 8, 10, 12, 14, 16, 18, and 20 are malicious clients, where the malicious client group performs the specified attack method.

**Dataset Setup**: In all three attack experiments, clients were set up with independently homogeneous data distributions (IID). For instance, in a 10-client experiment, each client has 6000 samples for MNIST and Fashion-MNIST. Each client has 5000 samples for CIFAR-10.

In particular, in the noise attack experiments, we set different ratios of noise data for the malicious client groups, in which for the MNIST dataset, the noise data ratios of clients 2, 4, 6, 8, 10, 12, 14, 16, 18, 20 are 0.91, 0.92, 0.93, 0.94, 0.95, 0.91, 0.92, 0.93, 0.94, 0.95 , respectively. For the Fasion-MNIST dataset, the noise data ratios of malicious clients are 0.75, 0.8, 0.85, 0.9, 0.95, 0.75, 0.8, 0.85, 0.9, 0.95 , respectively. For the CIFAR-10 dataset, they are 0.5, 0.55, 0.6, 0.65, 0.7, 0.5, 0.55, 0.6, 0.65, 0.7 , respectively.

To evaluate the performance of the client identification module, we process logit information in the following three steps:

(1) Vector Conversion: We gathered the logit information from the client and server. Then, we group the logit for each category and transform each subgroup into a one-dimensional vector.

(2) Cosine Similarity Calculation: For each category vector, we calculated the cosine similarity between the client’s category vectors and the server’s corresponding category vectors. This similarity measurement serves as a pivotal metric in our evaluation.

(3) Feature Value Calculation: We regard the cosine similarity values for the various categories as the feature values for each client.

**Figure 9:**
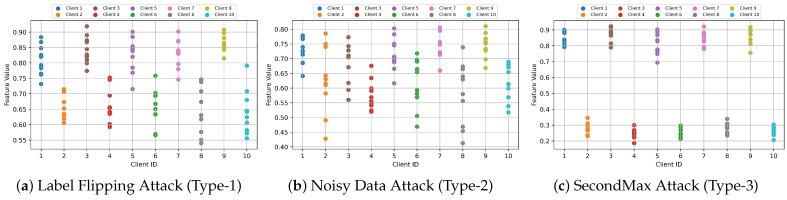
The feature values of each client in different attack scenarios.

We take the CIFAR-10 dataset as an example and apply this methodology to this dataset. We conduct the experiment on the three specified attack scenarios. The feature values for each client under these scenarios are illustrated in Figure 9. This figure reveals notable disparities in feature values between normal and malicious clients, particularly in Type-1 and Type-3 attack scenarios. In contrast, the Type-2 (noisy data attack) scenario demonstrates a variation in feature values across certain categories, though not as pronounced as in the other types.

After computing the feature values for each category of each client, we proceed with the following steps:

(1) Client Clustering: We take the cosine similarity value of each category as the feature values of each client. Then, we employ the K-means method to cluster clients into two subgroups.

(2) Accuracy Verification on Public Data: For each resulting cluster, we assess the accuracy of the logit on the public dataset. The subgroup exhibiting the highest accuracy is deemed the ’trusted client list,’ while the one with lower accuracy is classified as comprising malicious clients.

**Figure 10:**
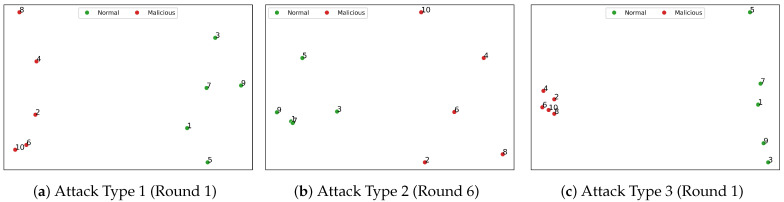
Clustering results for each client under different attack scenarios (10 clients).

We follow the steps above for clustering analysis, and the clustering outcomes are presented in Figure 10. In Attack Types 1 and 3, our method successfully identifies malicious clients from the first communication round. In later rounds, the difference between the feature values of normal and malicious clients will become larger and larger, and our method can more easily separate the client types. However, in Attack Type 2, our method requires up to six communication rounds to categorize the clients accurately. The initial rounds are marked by relatively low logit accuracy, which obscures the differences between normal and malicious clients. As a result, some malicious clients may initially be misclassified as normal. To mitigate this issue, we need to verify client logit accuracy further, as detailed in the fourth stage of our method. This additional step is crucial for ensuring accurate client categorization in scenarios where initial data may not be distinct enough for immediate classification.

#### 5.2.2. Experimental Analysis of Different Attack Scenarios

This experiment mainly evaluates our algorithm’s robustness across various attack scenarios. We benchmark our algorithm against five baseline methods, using two key performance metrics: the accuracy of the global logit on the public dataset and the average client model’s accuracy.

We tested the algorithms under predefined attack scenarios, adhering to our experimental configuration. As the FedDistill+ and FedHe methods do not necessitate a public dataset, our comparison of the accuracy of the global logitis is limited to the three algorithms (FedMD, FedDF, FedDistill). The experimental results are shown in Table 2. The experiment shows that our algorithms consistently outperform others across three datasets.

Notably, in Attack Types 1 and 2, the global logit accuracy of FedMD, FedDF, and FedDistill exhibited a significant decline. This observation suggests that these algorithms may not be as effective in mitigating the impact of Attack Types 1 and 2.

**Figure 11:**
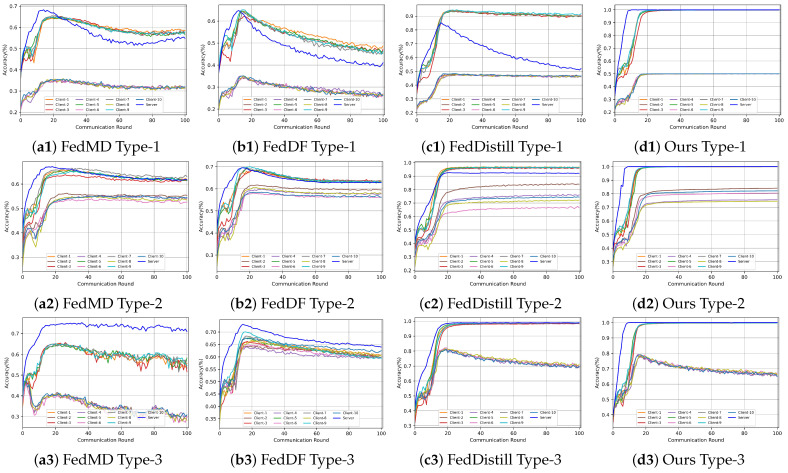
The accuracy of each client in the public dataset under different attack scenarios (10 clients).

To illustrate the influence of the malicious client’s logit accuracy on the overall global logit, we meticulously tracked the logit accuracy of each client across all communication rounds. The results are shown in Figure 11. We analyze the impact of different attack scenarios on each algorithm as follows:

Attack Type 1: In this scenario, malicious clients significantly compromised the global logit accuracy of methods such as FedMD, FedDF, and FedDistill. The primary reason for this degradation is that these methods do not filter out malicious clients during the fusion process of the global logit.

Attack Type 2: In this scenario, FedMD, FedDF, and FedDistill methods use a simplistic weighted calculation for fusing global logit. Consequently, they fail to discern low-quality model outputs, leading to a reduction in logit accuracy.

Attack Type 3: The FedMD and FedDF methods are particularly vulnerable to tampered logit information, resulting in diminished global knowledge accuracy.

Contrastingly, our method consistently maintains high accuracy across all three attack scenarios. This resilience stems from our method’s capability to exclude malicious information from the fusion process and our server’s function to detect and ensure the fusion of high-quality logit information.

Furthermore, we evaluated the impact of different algorithms on average client accuracy. To assess how malicious clients affect federated tasks, we also introduce the scenarios without malicious clients (denoted as ’normal type’). These results are detailed in Table 3. our algorithm demonstrates superior accuracy on both the Fashion-MNIST and CIFAR-10 datasets. On the MNIST dataset, our algorithm slightly trails behind the FedHe algorithm in Attack Type 1 and FedDistill in Attack Type 3. This outcome can be attributed to the relative simplicity of the MNIST dataset, where methods like FedDistill+ and FedHe, which regularize categories, tend to achieve higher accuracy.

**Figure 12:**
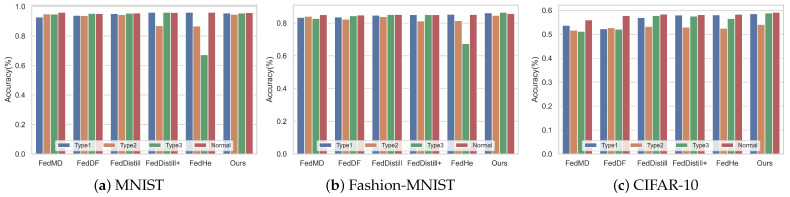
Average client accuracy for each algorithm in different attack scenarios.

We conducted a comparative analysis of different algorithms to evaluate their performance under various attack scenarios. we take the CIFAR-10 as an example, and the results are shown in Figure 12. Notably, algorithm performance varies significantly depending on the type of attack:

Attack Type 1: Both FedDistill+ and FedHe methods experienced a notable decrease in accuracy. This suggests that these methods may be more vulnerable or less equipped to handle the specific challenges posed by this attack type.

Attack Type 2: The FedMD and FedDF methods showed a marked decline in performance. This indicates that these methods, while possibly effective in other scenarios, struggle to maintain accuracy under the conditions of Attack Type 2.

**Figure 13:**
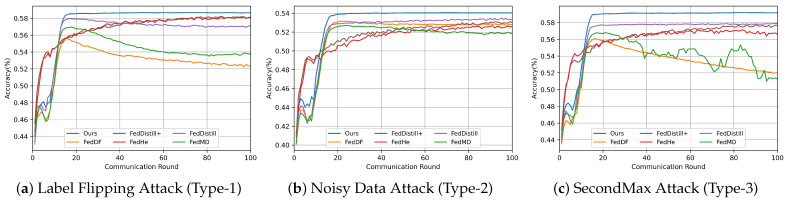
Average client accuracy for different attack scenarios (10 clients).

To illustrate how the accuracy of different algorithms fluctuates across communication rounds, we conducted an in-depth analysis using the CIFAR-10 dataset. We calculated the average client model accuracy at each communication round, as shown in Figure 13.

In Attack Types 1 and 3, the accuracy of the FedMD and FedDF algorithms showed a significant decrease with increasing communication rounds. This decline is attributed to their reliance on a simple weighted average method for global logit fusion. Such a method proves ineffective in excluding the erroneous logit information from malicious clients, thereby diminishing the overall accuracy of the global logit. As a result, clients utilizing this compromised information for local model training experience a reduction in their model’s accuracy.

Our algorithm consistently achieves the highest accuracy rates across all three attack scenarios. The strength of our method lies in its ability to prevent malicious information from influencing the model fusion process. Additionally, it empowers the server to fuse high-quality knowledge from the client selectively. This capability ensures that our approach remains stable and effective under various attack scenarios.

#### 5.2.3. Experimental Analysis of Different Data Distribution Scenarios

In this section, we focus on assessing each algorithm’s performance in different data distribution scenarios. The experimental setup is as follows:

**Federated Task Configuration**: We set different numbers of clients (10, 15, and 20) to participate in the federated task with 100 communication rounds. Each client completed one training epoch, while the server conducted two on the public dataset.

**Network Model Configuration**: We follow Table 1 of the previous section to set up the client-side and server-side models in each scenario.

**Dataset Division**: We divided each dataset using the Dirichlet method of the previous subsection. This involved creating three Non-IID data scenarios ( α={0.5, 1, 10} ) and one IID data distribution scenario.

Following these specified configurations, the experiments were conducted separately for each data distribution scenario. The accuracy results of each algorithm under these conditions are compiled and presented in Table 4.

The experiment demonstrates that our algorithms consistently achieve the highest accuracy across all data distribution scenarios on the three datasets. Notably, the performance of the FedMD and FedDF algorithms exhibits a decline in Non-IID scenarios (α={0.5, 1}) , a trend that is particularly pronounced in the CIFAR-10 dataset.

**Figure 14:**
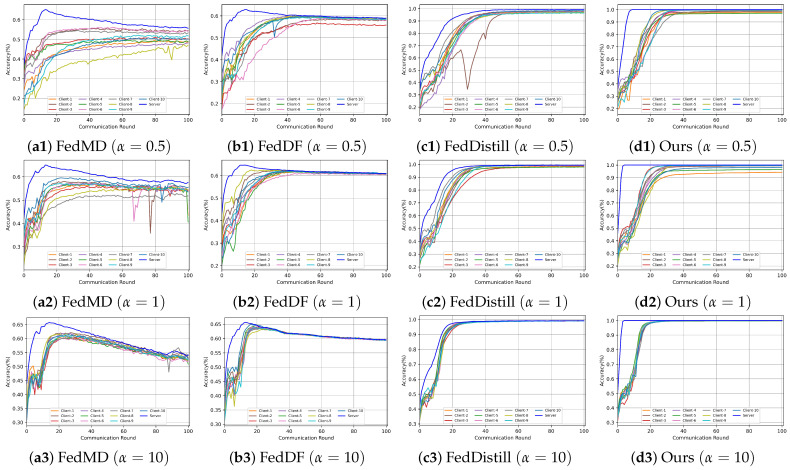
The accuracy of each client in a public dataset with different data distributions (10 clients).

To further explore the relationship between client and global logit accuracy, we meticulously tracked and analyzed the accuracy of each client and the global logit for every communication round, as shown in Figure 14. The accuracy of the FedMD and FedDF algorithms diminishes as the disparity in Non-IID data distribution increases. This trend underscores that the performance of algorithms relying on average weighting methods, such as FedMD and FedDF, is significantly hindered in Non-IID scenarios. In contrast, our algorithm and FedDistill demonstrate robustness against the variation in data distribution, maintaining consistent accuracy irrespective of the data scenario.

To verify the performance of each algorithm under different data distribution scenarios, we also calculated the average client model accuracy metrics, and the experimental results are shown in Table 5. The experiment results show that our algorithms obtain the highest values on both Fashion-MNIST and CIFAR-10 datasets. The FedDistill+ and FedHe algorithms show a serious drop in accuracy in Non-IID ( α={0.5, 1} ) scenarios, which indicates that these two algorithms do not work stably in Non-IID scenarios.

**Figure 15:**
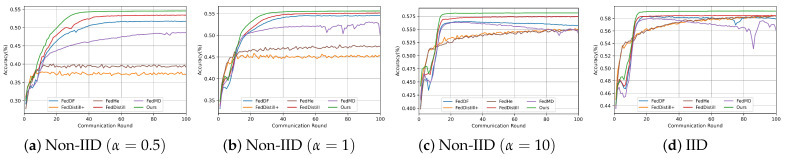
Average client accuracy for different data distribution scenarios (10 clients).

To compare the accuracy of each algorithm on different rounds, we take the CIFAR-10 dataset as an example and compare the accuracy of five algorithms under different data distribution scenarios. The results are shown in Figure 15. The experiments show that our algorithms obtain the highest accuracy under different data scenarios.

We utilized the CIFAR-10 dataset to assess the effectiveness of the adaptive knowledge distillation method. This involved monitoring the weight values assigned to each category over each communication round. We adjusted the hyperparameters for different data distribution scenarios for optimal performance. Specifically, in Non-IID scenarios ( α={0.5, 1} ), we set the β parameter to 0.8. For the Non-IID scenario ( α=10 ) and the IID scenario, we set the β parameter to 0.75.

**Figure 16:**
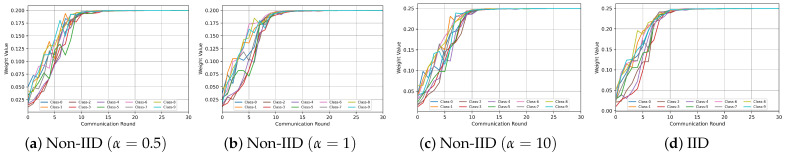
Weight allocation for each category in different data scenarios (10 clients).

The weight values of each category for different data scenarios are shown in Figure 16. The results show that the weight values of each scenario no longer change after 15 communication rounds. This is because when our communication rounds reach a certain number of rounds, our algorithm has fully integrated the knowledge of each client. At the same time, the prediction of the samples of each category in the public dataset reaches the best accuracy, so after 15 rounds, the weight values of each category no longer change.

#### 5.2.4. Performance Validation of the FedTKD Framework

We evaluated the computational efficiency of four main modules within our federated learning framework. These modules are Client-Side Identification, Server-Side Model Training, Global Logit Information Computation, and Category Weight Computation.

**Figure 17:**
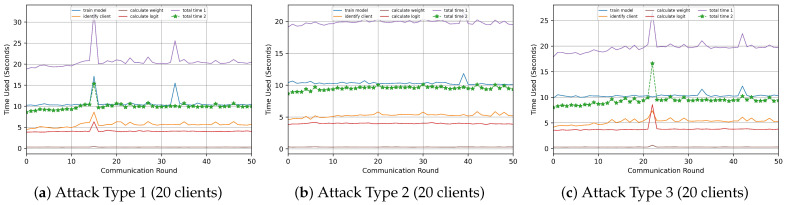
Computation time for each model per communication round.

Experimental Setup: We set the number of communication rounds in the federated task to 50 for this evaluation. We meticulously recorded the computation time of each module across every communication round under each attack scenario. These results are presented in Figure 17.

Experimental Analysis: The experimental result reveals that the computation times of each round for each module are generally consistent, with only occasional variations. These inconsistencies are attributed to the high-performance workstations used for the experiments, which ran other tasks concurrently. This multitasking environment led to fluctuating resource allocation, resulting in sporadic peaks in computation time.

To gain a more comprehensive understanding of computational efficiency, we also calculated the average computation time for each module throughout the entire federated task. The detailed results are tabulated in Table 6. We differentiate between ‘total time 1’ and ‘total time 2’. The ‘total time 1’ is the aggregate time for all modules. The ‘total time 2’ is the cumulative time of all modules, excluding the server-side training model module.

The experiment shows that the number of clients does not influence the training model and logit calculation computation times. The training model times depend on network parameters, epochs, and the number of samples. The logit calculation time depends on the number of samples in the public dataset. The computation time of these two modules does not increase with the number of nodes because the factors affecting these modules are independent of the number of nodes. The time required for client identification and weight calculation increases with the number of clients. For server-side computational efficiency, the ‘total time 2’ is more relevant as server-side model training is independent of client-side tasks. We assume the server has sufficient computational resources to pre-train the model before receiving the client’s logit. Thus, the ‘total time 2’ can reflect the efficiency of our algorithm in processing client logit messages. Notably, in scenarios with 20 clients, our framework can complete essential steps in under 10 s, which is crucial for the real-time detection of client type.

## 6. Conclusions

To address the issue of trustworthy computing and information fusion in heterogeneous federated environments, we have designed a trustworthy federated learning framework (FedTKD). This framework encompasses several key functions. Firstly, we proposed a client recognition method based on logit features to exclude malicious clients from participating in global information fusion. Additionally, we proposed a reliable global logit fusion method to ensure high-quality information fusion. Finally, we proposed an adaptive knowledge distillation method to improve the accuracy of knowledge transfer from the server side to the client side. We conducted experiments in various attack scenarios, including logit tag flipping and low-quality information fusion scenarios, to assess the reliability and robustness of FedTKD. Furthermore, we have evaluated the algorithm’s performance under different data distribution scenarios and compared it with five baseline algorithms. The results demonstrate that our algorithms outperform others in various circumstances.

However, it is important to note that our approach requires a common dataset shared between the server and clients, which should include samples of all types. In practical scenarios, creating such a comprehensive common dataset can be challenging. Therefore, our future research will investigate how to achieve trustworthy heterogeneous federated learning when only a subset of sample types is available. We also plan to explore a wider range of client attacks to expand the applicability of our method. Additionally, we intend to deploy FedTKD in a distributed environment to assess and optimize its real-world performance.

## Figures and Tables

**Table 1:** Network configuration in heterogeneous environments.

ID	Conv-1	Conv-2	Conv-3	Conv-4	Conv-5	Conv-6	FC
Server	K = 3, S = 1	C2d (16, 16)	C2d (16, 32)	C2d (32, 64)	C2d (32, 64)	C2d (54, 128)	Line (512, 512)
	Out = 16	C2d (16, 16)	C2d (32, 32)	C2d (64, 64)	C2d (64, 64)	C2d (256, 512)	Line (512, 10)
1, 2, 11, 16	K = 3, S = 1	C2d (16, 16)	C2d (16, 32)	C2d (32, 64)	C2d (32, 64)	C2d (54, 128)	Line (512, 512)
	Out = 16	C2d (16, 16)	C2d (32, 32)	C2d (64, 64)	C2d (64, 64)	C2d (256, 512)	Line (512, 10)
3, 4, 12, 17	K = 3, S = 1	C2d (16, 16)	C2d (16, 32)	C2d (32, 64)	C2d (32, 64)	C2d (64, 128)	Line (256, 256)
	Out = 16	C2d (16, 16)	C2d (32, 32)	C2d (64, 64)	C2d (64, 64)	C2d (256, 256)	Line (256, 10)
5, 6, 13, 18	K = 3, S = 1	C2d (16, 16)	C2d (16, 32)	C2d (32, 64)	C2d (32, 64)	-	Line (512, 128)
	Out = 16	C2d (16, 16)	C2d (32, 32)	C2d (64, 64)	C2d (64, 64)	-	Line (128, 10)
7, 8, 14, 19	K = 3, S = 1	C2d (16, 16)	C2d (16, 32)	C2d (32, 64)	-	-	Line (1024, 128)
	Out = 16	C2d (16, 16)	C2d (32, 32)	C2d (64, 64)	-	-	Line (128, 10)
9, 10, 15, 20	K = 3, S = 1	C2d (16, 16)	C2d (32, 32)	C2d (64, 64)	-	-	Line (2048, 256)
	Out = 16	C2d (16, 16)	C2d (32, 32)	C2d (64, 128)	-	-	Line (256, 10)

**Table 2:** The global logit accuracy of each algorithm in different attack types.

Number	Method	MNIST			Fashion-MNIST			CIFAR-10		
		Type-1	Type-2	Type-3	Type-1	Type-2	Type-3	Type-1	Type-2	Type-3
10 clients	FedMD	0.54	0.961	0.968	0.577	0.894	0.901	0.548	0.617	0.709
	FedDF	0.75	0.953	0.973	0.706	0.897	0.915	0.41	0.628	0.639
	FedDistill	0.83	0.996	0.996	0.796	0.964	0.966	0.52	0.92	0.988
	Ours	**0.997**	**0.998**	**0.998**	**0.996**	**0.998**	**0.996**	**0.997**	**0.996**	**0.998**
15 clients	FedMD	0.846	0.945	0.949	0.684	0.867	0.884	0.556	0.601	0.676
	FedDF	0.835	0.941	0.958	0.746	0.868	0.886	0.446	0.595	0.634
	FedDistill	0.891	0.979	0.978	0.815	0.913	0.911	0.715	0.861	0.992
	Ours	**0.988**	**0.986**	**0.992**	**0.936**	**0.939**	**0.932**	**0.998**	**0.996**	**0.997**
20 clients	FedMD	0.837	0.936	0.943	0.675	0.885	0.869	0.528	0.583	0.663
	FedDF	0.835	0.941	0.958	0.676	0.855	0.869	0.446	0.564	0.601
	FedDistill	0.891	0.979	0.978	0.787	0.898	0.903	0.611	0.851	0.992
	Ours	**0.989**	**0.989**	**0.99**	**0.932**	**0.937**	**0.931**	**0.998**	**0.993**	**0.998**

**Table 3:** Average client model accuracy of each algorithm in different attack scenarios.

Number	Method	MNIST				Fashion-MNIST				CIFAR-10			
		Type-1	Type-2	Type-3	Normal	Type-1	Type-2	Type-3	Normal	Type-1	Type-2	Type-3	Normal
10 clients	FedMD	0.928	0.945	0.948	0.961	0.835	0.841	0.828	0.851	0.537	0.517	0.512	0.56
	FedDF	0.939	0.938	0.953	0.952	0.837	0.841	0.847	0.849	0.523	0.527	0.521	0.578
	FedDistill	0.952	0.945	0.954	0.956	0.848	0.839	0.852	0.852	0.57	0.533	0.578	0.584
	FedDistill+	0.96	0.869	**0.96**	0.959	0.851	0.812	0.851	0.851	0.58	0.53	0.576	0.582
	FedHe	**0.961**	0.867	0.673	**0.961**	0.853	0.815	0.674	0.852	0.581	0.525	0.566	0.583
	Ours	0.955	**0.947**	0.955	0.958	**0.862**	**0.847**	**0.855**	**0.858**	**0.586**	**0.541**	**0.589**	**0.592**
15 clients	FedMD	0.942	0.936	0.927	0.949	0.822	0.819	0.823	0.835	0.513	0.501	0.505	0.547
	FedDF	0.935	0.927	0.941	0.939	0.746	0.816	0.826	0.828	0.446	0.492	0.503	0.546
	FedDistill	0.942	0.934	0.943	0.945	0.823	0.816	0.826	0.827	0.552	0.518	0.553	0.557
	FedDistill+	0.947	0.832	**0.947**	0.947	0.838	0.784	0.84	0.841	0.528	0.484	0.518	0.528
	FedHe	0.941	0.779	0.938	**0.95**	0.842	0.789	0.834	0.843	0.534	0.492	0.528	0.536
	Ours	**0.947**	**0.939**	0.946	0.945	**0.843**	**0.821**	**0.842**	**0.848**	**0.564**	**0.528**	**0.56**	**0.562**
20 clients	FedMD	0.936	0.928	0.912	**0.942**	0.813	0.809	0.811	0.828	0.482	0.481	0.487	0.525
	FedDF	0.923	0.921	0.934	0.932	0.815	0.808	0.818	0.818	0.464	0.474	0.483	0.527
	FedDistill	0.942	0.934	0.939	0.938	0.817	0.813	0.821	0.817	0.531	0.502	0.541	0.54
	FedDistill+	0.938	0.787	0.937	0.937	0.832	0.764	0.833	0.831	0.497	0.457	0.496	0.498
	FedHe	**0.95**	0.829	0.938	0.941	0.836	0.768	0.825	0.834	0.534	0.492	0.528	0.506
	Ours	0.942	**0.934**	**0.941**	0.941	**0.843**	**0.821**	**0.838**	**0.835**	**0.545**	**0.532**	**0.545**	**0.546**

**Table 4:** The global logit accuracy of each algorithm in different data distribution scenarios.

Number	Method	MNIST				Fashion-MNIST				CIFAR-10			
		α=0.5	α=1	α=10	IID	α=0.5	α=1	α=10	IID	α=0.5	α=1	α=10	IID
**10 clients**	FedMD	0.95	0.958	0.961	0.974	0.871	0.875	0.884	0.91	0.556	0.576	0.541	0.66
	FedDF	0.948	0.947	0.949	0.972	0.866	0.878	0.884	0.905	0.589	0.609	0.595	0.726
	FedDistill	0.993	0.993	0.997	0.997	0.927	0.946	0.953	0.96	0.991	0.994	0.99	0.994
	Ours	**0.997**	**0.998**	**0.998**	**0.998**	**0.997**	**0.996**	**0.997**	**0.998**	**0.995**	**0.996**	**0.998**	**0.999**
**15 clients**	FedMD	0.937	0.945	0.941	0.957	0.855	0.862	0.872	0.883	0.57	0.576	0.583	0.669
	FedDF	0.929	0.937	0.948	0.951	0.859	0.856	0.876	0.882	0.582	0.572	0.575	0.665
	FedDistill	0.966	0.967	0.969	0.976	0.889	0.895	0.901	0.907	0.983	0.991	0.993	0.995
	Ours	**0.987**	**0.988**	**0.989**	**0.992**	**0.921**	**0.932**	**0.934**	**0.935**	**0.996**	**0.998**	**0.997**	**0.998**
**20 clients**	FedMD	0.928	0.936	0.941	0.951	0.855	0.86	0.862	0.875	0.546	0.55	0.568	0.645
	FedDF	0.929	0.931	0.938	0.94	0.851	0.86	0.861	0.872	0.551	0.554	0.543	0.631
	FedDistill	0.965	0.967	0.97	0.971	0.877	0.884	0.893	0.899	0.99	0.991	0.993	0.997
	Ours	**0.987**	**0.989**	**0.989**	**0.991**	**0.932**	**0.932**	**0.927**	**0.935**	**0.992**	**0.996**	**0.998**	**0.998**

**Table 5:** Average client model accuracy of each algorithm in different data distribution scenarios.

Number	Method	MNIST				Fashion-MNIST				CIFAR-10			
		α=0.5	α=1	α=10	IID	α=0.5	α=1	α=10	IID	α=0.5	α=1	α=10	IID
10 clients	FedMD	0.931	0.95	**0.959**	**0.961**	0.781	0.825	0.848	0.851	0.486	0.499	0.542	0.56
	FedDF	0.933	0.938	0.948	0.952	0.799	0.824	0.845	0.849	0.517	0.545	0.557	0.578
	FedDistill	0.938	0.946	0.953	0.956	0.777	0.827	0.842	0.852	0.534	0.551	0.574	0.584
	FedDistill+	0.799	0.865	0.956	0.959	0.703	0.786	0.845	0.851	0.371	0.453	0.549	0.582
	FedHe	0.788	0.846	0.955	**0.961**	0.698	0.762	0.846	0.852	0.391	0.474	0.549	0.583
	Ours	**0.956**	**0.958**	0.957	0.958	**0.812**	**0.832**	**0.853**	**0.858**	**0.545**	**0.565**	**0.583**	**0.592**
15 clients	FedMD	0.901	0.928	**0.944**	0.949	0.751	0.788	0.833	0.835	0.44	0.489	0.528	0.547
	FedDF	0.902	0.92	0.935	0.939	0.766	0.791	0.822	0.828	0.505	0.509	0.531	0.546
	FedDistill	0.913	0.925	0.939	0.945	0.766	0.793	0.821	0.827	0.505	0.528	0.548	0.557
	FedDistill+	0.731	0.838	0.939	0.947	0.657	0.764	0.832	0.841	0.336	0.39	0.497	0.528
	FedHe	0.705	0.859	0.943	**0.95**	0.646	0.728	0.831	0.843	0.354	0.413	0.506	0.536
	Ours	**0.924**	**0.934**	0.937	0.945	**0.77**	**0.802**	**0.836**	**0.848**	**0.528**	**0.542**	**0.555**	**0.562**
20 clients	FedMD	0.884	0.909	0.937	**0.942**	0.734	0.781	0.823	0.828	0.426	0.463	0.513	0.525
	FedDF	0.899	0.913	0.927	0.932	0.766	0.787	0.821	0.818	0.485	0.497	0.514	0.527
	FedDistill	0.907	0.919	0.934	0.938	0.751	0.795	0.822	0.817	0.499	0.515	0.533	0.54
	FedDistill+	0.686	0.843	0.929	0.937	0.619	0.74	0.822	0.831	0.314	0.379	0.476	0.498
	FedHe	0.673	0.823	0.933	0.941	0.657	0.735	0.824	0.834	0.336	0.373	0.483	0.506
	Ours	**0.918**	**0.924**	**0.939**	0.941	**0.769**	**0.796**	**0.83**	**0.835**	**0.519**	**0.522**	**0.54**	**0.546**

**Table 6:** Average computation time for each module on the server side (seconds).

Number	Attack	Train Model	Identify Client	Calculate Logit	Calculate Weight	Total Time 1	Total Time 2
10 clients	Type-1	10.112	2.802	0.298	2.081	15.294	5.181
	Type-2	10.095	2.724	0.296	2.039	15.155	5.06
	Type-3	10.196	2.679	0.301	1.932	15.108	4.912
15 clients	Type-1	10.3	4.017	0.292	2.951	17.56	7.26
	Type-2	10.497	3.964	0.297	3.005	17.764	7.267
	Type-3	10.26	3.885	0.293	2.807	17.215	6.955
20 clients	Type-1	10.625	5.611	0.298	4.069	20.604	9.978
	Type-2	10.321	5.259	0.295	3.984	19.86	9.539
	Type-3	10.337	5.278	0.299	3.826	19.74	9.403

## Data Availability

Data are contained within the article.

## References

[1] McMahan B., Moore E., Ramage D., Hampson S., y Arcas B.A. Communication-efficient learning of deep networks from decentralized data. Proceedings of the Artificial Intelligence and Statistics.

[2] Karimireddy S.P., Kale S., Mohri M., Reddi S., Stich S., Suresh A.T. Scaffold: Stochastic controlled averaging for federated learning. Proceedings of the International Conference on Machine Learning.

[3] Li T., Sahu A.K., Zaheer M., Sanjabi M., Talwalkar A., Smith V. (2020). Federated optimization in heterogeneous networks. Proc. Mach. Learn. Syst..

[4] Xie C., Koyejo S., Gupta I. (2019). Asynchronous federated optimization. arXiv.

[5] Hinton G., Vinyals O., Dean J. (2015). Distilling the knowledge in a neural network. arXiv.

[6] Fukuda T., Suzuki M., Kurata G., Thomas S., Cui J., Ramabhadran B. Efficient Knowledge Distillation from an Ensemble of Teachers. Proceedings of the Interspeech.

[7] Li D., Wang J. (2019). Fedmd: Heterogenous federated learning via model distillation. arXiv.

[8] Lin T., Kong L., Stich S.U., Jaggi M. (2020). Ensemble distillation for robust model fusion in federated learning. Adv. Neural Inf. Process. Syst..

[9] Jiang D., Shan C., Zhang Z. Federated learning algorithm based on knowledge distillation. Proceedings of the 2020 International Conference on Artificial Intelligence and Computer Engineering (ICAICE).

[10] Zhu Z., Hong J., Zhou J. Data-free knowledge distillation for heterogeneous federated learning. Proceedings of the International Conference on Machine Learning.

[11] Zhang L., Shen L., Ding L., Tao D., Duan L.Y. Fine-tuning global model via data-free knowledge distillation for non-iid federated learning. Proceedings of the IEEE/CVF Conference on Computer Vision and Pattern Recognition.

[12] Zhang Z., Shen T., Zhang J., Wu C. (2022). Feddtg: Federated data-free knowledge distillation via three-player generative adversarial networks. arXiv.

[13] Lu Q., Zhu L., Xu X., Whittle J., Xing Z. Towards a roadmap on software engineering for responsible AI. Proceedings of the 1st International Conference on AI Engineering: Software Engineering for AI.

[14] Lu Q., Zhu L., Xu X., Whittle J. (2023). Responsible-AI-by-design: A pattern collection for designing responsible AI systems. IEEE Softw..

[15] Chen L., Zhang W., Xu L., Zeng X., Lu Q., Zhao H., Chen B., Wang X. A Federated Parallel Data Platform for Trustworthy AI. Proceedings of the 2021 IEEE 1st International Conference on Digital Twins and Parallel Intelligence (DTPI).

[16] Wang J., Liu Q., Liang H., Joshi G., Poor H.V. (2020). Tackling the objective inconsistency problem in heterogeneous federated optimization. Adv. Neural Inf. Process. Syst..

[17] Li Q., He B., Song D. Model-contrastive federated learning. Proceedings of the IEEE/CVF Conference on Computer Vision and Pattern Recognition.

[18] Seo H., Park J., Oh S., Bennis M., Kim S.L. (2022). Federated Knowledge Distillation. Machine Learning and Wireless Communications.

[19] Chen H., Vikalo H. (2023). The Best of Both Worlds: Accurate Global and Personalized Models through Federated Learning with Data-Free Hyper-Knowledge Distillation. arXiv.

[20] Li S., Cheng Y., Wang W., Liu Y., Chen T. (2020). Learning to detect malicious clients for robust federated learning. arXiv.

[21] Chen L., Dong C., Qiao S., Huang Z., Nie Y., Hou Z., Tan C. (2023). FedDRL: A Trustworthy Federated Learning Model Fusion Method Based on Staged Reinforcement Learning. arXiv.

[22] Blanchard P., El Mhamdi E.M., Guerraoui R., Stainer J. Machine learning with adversaries: Byzantine tolerant gradient descent. Proceedings of the Advances in Neural Information Processing Systems 30 (NIPS 2017).

[23] Chen L., Zhao D., Tao L., Zeng X., Tan C. (2024). A Credible and Fair Federated Learning Framework Based on Blockchain. IEEE Trans. Artif. Intell..

[24] Yin D., Chen Y., Kannan R., Bartlett P. Byzantine-robust distributed learning: Towards optimal statistical rates. Proceedings of the International Conference on Machine Learning.

[25] Li S., Ngai E.C.H., Voigt T. (2023). An Experimental Study of Byzantine-Robust Aggregation Schemes in Federated Learning. arXiv.

[26] Karimireddy S.P., He L., Jaggi M. Learning from history for byzantine robust optimization. Proceedings of the International Conference on Machine Learning.

[27] Zhang J., Ge C., Hu F., Chen B. (2021). RobustFL: Robust federated learning against poisoning attacks in industrial IoT systems. IEEE Trans. Ind. Inform..

[28] Wang Y., Xie L., Liu X., Yin J.L., Zheng T. Model-agnostic adversarial example detection through logit distribution learning. Proceedings of the 2021 IEEE International Conference on Image Processing (ICIP).

[29] Cheng S., Wu J., Xiao Y., Liu Y. (2021). Fedgems: Federated learning of larger server models via selective knowledge fusion. arXiv.

[30] Zhang H., Chen D., Wang C. Confidence-aware multi-teacher knowledge distillation. Proceedings of the ICASSP 2022—2022 IEEE International Conference on Acoustics, Speech and Signal Processing (ICASSP).

[31] He Y., Chen Y., Yang X., Zhang Y., Zeng B. Class-wise adaptive self distillation for heterogeneous federated learning. Proceedings of the 36th AAAI Conference on Artificial Intelligence.

[32] Lukasik M., Bhojanapalli S., Menon A.K., Kumar S. (2021). Teacher’s pet: Understanding and mitigating biases in distillation. arXiv.

[33] Chan Y.H., Ngai E.C. Fedhe: Heterogeneous models and communication-efficient federated learning. Proceedings of the 2021 17th International Conference on Mobility, Sensing and Networking (MSN).

